# Identification and characterization of putative biomarkers and therapeutic axis in Glioblastoma multiforme microenvironment

**DOI:** 10.3389/fcell.2023.1236271

**Published:** 2023-07-19

**Authors:** Smita Kumari, Pravir Kumar

**Affiliations:** Molecular Neuroscience and Functional Genomics Laboratory, Department of Biotechnology, Delhi Technological University, Delhi, India

**Keywords:** non-cellular secretory components, computational biology, E2 conjugating enzymes, acetylation, Glioblastoma multiforme, tumor microenvironment, protein kinases

## Abstract

Non-cellular secretory components, including chemokines, cytokines, and growth factors in the tumor microenvironment, are often dysregulated, impacting tumorigenesis in Glioblastoma multiforme (GBM) microenvironment, where the prognostic significance of the current treatment remains unsatisfactory. Recent studies have demonstrated the potential of post-translational modifications (PTM) and their respective enzymes, such as acetylation and ubiquitination in GBM etiology through modulating signaling events. However, the relationship between non-cellular secretory components and post-translational modifications will create a research void in GBM therapeutics. Therefore, we aim to bridge the gap between non-cellular secretory components and PTM modifications through machine learning and computational biology approaches. Herein, we highlighted the importance of BMP1, CTSB, LOX, LOXL1, PLOD1, MMP9, SERPINE1, and SERPING1 in GBM etiology. Further, we demonstrated the positive relationship between the E2 conjugating enzymes (Ube2E1, Ube2H, Ube2J2, Ube2C, Ube2J2, and Ube2S), E3 ligases (VHL and GNB2L1) and substrate (HIF1A). Additionally, we reported the novel HAT1-induced acetylation sites of Ube2S (K211) and Ube2H (K8, K52). Structural and functional characterization of Ube2S (8) and Ube2H (1) have identified their association with protein kinases. Lastly, our results found a putative therapeutic axis HAT1-Ube2S(K211)-GNB2L1-HIF1A and potential predictive biomarkers (CTSB, HAT1, Ube2H, VHL, and GNB2L1) that play a critical role in GBM pathogenesis.

## Graphical Abstract

**Figure:**
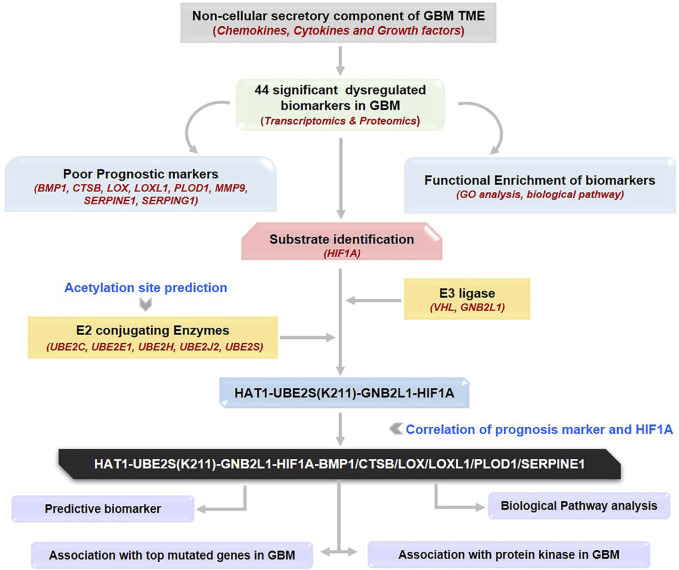
The workflow illustrates the process of identification of a novel therapeutic axis for targeting the GBM microenvironment. GBM: Glioblastoma multiforme, TME: Tumor microenvironment.

## Highlights

• BMP1, CTSB, LOX, LOXL1, PLOD1, MMP9, SERPINE1, and SERPING1 are linked with poor prognosis in GBM patients.
• CTSB, HAT1, Ube2H, VHL, and GNB2L1 are predictive markers for GBM therapies.
• The poor prognostic markers BMP1, CTSB, LOX, LOXL1, PLOD1, and SERPINE1 were positively linked with HIF1A.
• Ube2C (18, K33); Ube2E1 (K43); Ube2H (K8, K52); Ube2J2 (K64, K88); Ube2S (K198, K210, K211, K215, K216) as putative acetylated sites.
• Ube2H (K8, K52) and Ube2S (K211) are associated with overexpressed HAT1 enzymes in GBM.
• HAT1-Ube2S(K211)-GNB2L1-HIF1A-BMP1/CTSB/LOX/LOXL1/PLOD1/SERPINE1 as a novel therapeutic axis in GBM.

## 1 Introduction

*Glioblastoma* multiforme (*GBM*) is the most prevalent and fatal brain tumor with a poor prognosis. The clinical prognosis is still lacking despite several approved therapies for GBM, including surgery, radiation, and chemotherapy (Miller et al., 2021). The possible causes are the extensively invasive nature of GBM cells, the chemo- and radio-resistance, the high degree of vascularization, heterogeneity, and reduction of chemotherapeutic drugs effusion due to the blood-brain barrier (BBB), and heterogeneity of tumor microenvironment (TME). Further, the extracellular matrix (ECM) structural proteins are among the non-cellular components of the TME that are released by tumor or stromal cells or extravasated from the intravascular compartments other than cytokines, chemokines, and growth factors (Patel et al., 2018). Additionally, ECM structural proteins impact the development of all blood cells and other cells that support the body’s inflammatory and immunological reactions, which promote anti-cancer behavior (Baghban et al., 2020). The use of non-cellular secretory components as possible treatment targets and biomarker tools is now being investigated in several pre-clinical and clinical studies (Bridge et al., 2018; Liu C. et al., 2021). Cytokine expression patterns in GBM are distinctive, and aberrations in cytokine expression have been linked to gliomagenesis. The complex cytokine network in the diverse microenvironment facilitates interactions between the tumor cells, healthy brain cells, immune cells, and stem cells within the heterogeneous milieu of the GBM (Zhu et al., 2012). In addition, chemokines recruit different immune cell populations in TME by binding with their receptors. For instance, microglia cells implicated in their recruitment at the site of inflammation possess elevated amounts of CCR1 expression. These affect tumor growth, metastasis, the transition from low to high-grade gliomas, and treatment outcomes (Zeren et al., 2023). Another study demonstrates that the recurrence of GBM pathogenicity occurs when neural stem cells crosstalk with microglial cells (Dai et al., 2023). Moreover, studies have shown that post-translational modifications (PTMs), namely, methylation, acetylation, glycosylation, and ubiquitination of chemokines and cytokines, influence biological activities, inflammatory responses, and inflammasome-dependent innate immune responses through modifying the protein stability, structure, and sequence (Liu J. et al., 2016; Vanheule et al., 2018). A recent study by McCornack et al. (2023) discussed the significance of histone acetylation and methylation along with the consequences of targeted suppression of these enzymes by therapy in GBM (McCornack et al., 2023). Moreover, another study mentioned addressed the crucial role of histone acetylation in determining cell fate (Liu et al., 2023). Further, the exploration of new therapeutic interventions requires a thorough understanding of pathways relevant to GBM (Gallego-Perez et al., 2016). Additionally, protein kinases serve a crucial role in the signaling processes that regulate the traits of malignant cells, thereby making them valuable targets for therapeutic intervention in the management of cancer through the uptake of glucose, signaling modulation, epigenetic modifications, and progression of the cell cycle (Pang et al., 2022). Moreover, a variety of non-cellular secretory components of TME, including hormones, growth factors, chemokines, and cytokines bind to receptor tyrosine kinase and initiate downstream signaling, such as MAPK, PI3K/Ras that results in the proliferation and survival of tumor cells (Alexandru et al., 2020). EGFR signaling crosstalk with other major oncogenic signaling cascades, such as PI3K/protein kinase B (Akt)/mTOR pathway and MAPK pathway (Ramaiah and Kumar, 2021). However, in various cancers, protein kinase also controls TME and its constituent components. For example, in GBM tumor cells, IL-1β induces an HIF1A/IL-1β autocrine loop via activating Wnt-1 and RAS, which both contribute to the increase of HIF-1A (Chen et al., 2022). In contrast, IL-1β also stimulates the p38 MAPK-activated protein kinase 2-human antigen R (HuR), TLR-4, and other inflammatory-associated signaling pathways, which considerably enhance the levels of IL-6 and IL-8 in GBM tumor cells, eventually leading to an inflammatory TME in support of GBM invasion and growth (Gurgis et al., 2015). In addition, Cytokines, such as CCL5, was associated with intracellular calcium elevation. The activation of Akt and Ca^2+^/calmodulin-dependent protein kinase II (CaMKII) in GBM cells controlled the migratory and invasive activities (Yu-Ju Wu et al., 2020). Further, Tyrosine kinase inhibitors (TKI) and other kinase inhibitors (such as SI113) alone or in combination with other drugs/therapy have the potential to manage GBM by overcoming limitations such as BBB penetration, adaptation to altered signaling pathways, and heterogeneity of GBM cells (Alexandru et al., 2020) (Kim and Ko, 2020).

Moreover, histone acetyltransferases (HATs), besides histones, acetylates a variety of non-histone substrates, and thus, referred to as lysine acetyltransferases that play an essential function in normal and malignant hematopoiesis (Sun et al., 2015). Recent studies demonstrated that abnormally high histone acetylation levels could trigger chromatin-based mechanisms that promote tumorigenesis and malignant transformation. Further, it is interesting to note that most acetylated non-histone proteins are essential for immunological processes, tumorigenesis, and cancer cell growth (Spange et al., 2009). Evidence that lysine acetylation modification affects the lysosomal clearance of specific substrates and proteasomal degradation by either inhibiting or enhancing polyubiquitination (Narita et al., 2019). Additionally, studies have found that the UPS system degrades HIF1A after interacting with von Hippel–Lindau protein (pVHL) under normoxia, mediating its ubiquitination. For instance, Jeong et al. (2002) found that acetylation at specific lysine residues of HIF1A enhances its interaction with pVHL and its subsequent ubiquitination and degradation (Jeong et al., 2002). Likewise, acetylated retinoblastoma (Rb) recruits MDM2, an E3 ligase, and mutation in its acetylation hotspots is linked with an increased risk of breast cancer (Ullah et al., 2022). Acetylation has been studied extensively in proteosomes, Ub, E1, and E3 ligase, but few have in E2s.

**FIGURE 1:**
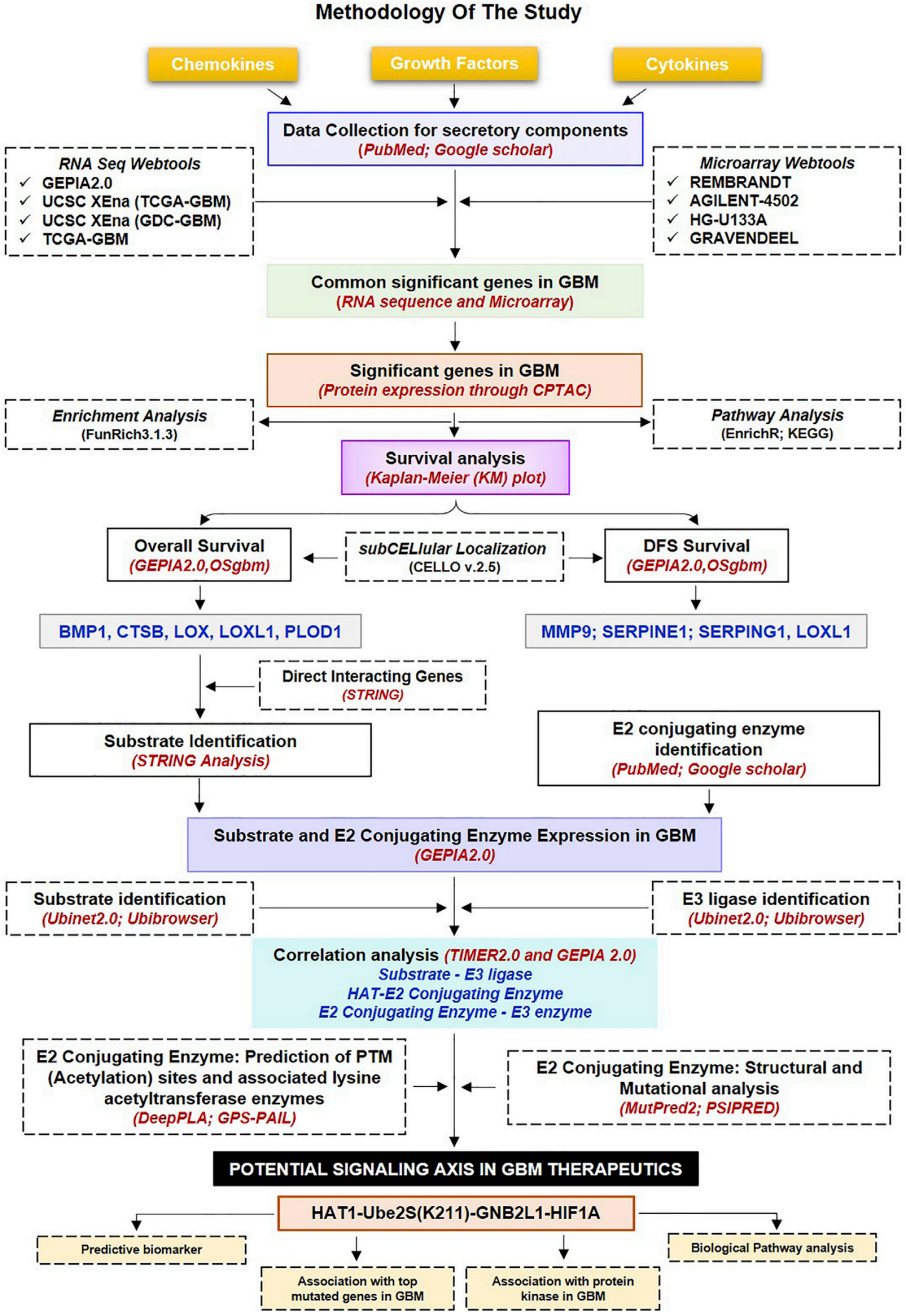
Methodology used in the current study: Workflow and steps considered along with the datasets collected and processed to identify prognostic and predictive markers in GBM. The expression of non-cellular secretory components (cytokines, chemokines, and growth factors) was examined in GBM transcriptome and proteomic data before the Kaplain-Meir plot was used to find prognostic markers. In addition, a common protein has been found that is directly associated with prognostic indicators; of these, two have the ability to function as substrates in the UPS system, and only HIF1A was elevated in GBM. Additionally, putative E3 ligases and E2s that are linked to HIF1A have been found. Additionally, a correlation study was done between prognostic markers, HIF1A, E3 ligase, E2s, and HAT enzymes. Further, a potential acetylation site on the lysine residues of E2s was found. The figure highlights the involvement of the acetylation mechanism, E2 conjugating enzymes, and E3 ligase’s finding novel therapeutic axis in GBM indication. Furthermore, a characterisation investigation of the suggested treatment axis was carried out. GBM: Glioblastoma Multiforme; E2s E2 conjugating enzymes, UPS: Ubiquitin proteasome systems.

Hence, the current study was conducted to understand better how acetylation affects E2s, which will fill the gap between UPS and acetylation modification and its impact on microenvironmental secretory protein regulations. Herein, we aim to identify novel therapeutic targets in GBM, including HATs, E1, E2s, and E3 ligases and substrates, as well as possible acetylation sites on lysine residues of E2 conjugating enzymes (E2s). We also systematically investigate the prognostic and predictive relevance of non-cellular secretory elements, such as chemokines, cytokines, and growth factors in GBM, and offer a model for clinical diagnosis. In addition, we have also established the correlation between biomarkers and dysregulated protein kinases in GBM. For the first time, we have looked at the involvement of E2s and how PTM, particularly acetylation, affects these enzymes. In typically, researchers always target substrate or E3 ligase. Figure 1 provides a quick overview of our analytical methodology, which adheres to the norms in bioinformatics investigations. We investigated the wide-ranging functions of non-cellular secretory components in the GBM microenvironment using the cancer genome atlas (TCGA) data. Hence, in-depth information about the expression of the whole family of secretory components and insights into the role of acetylation modification in UPS systems in GBM were provided by the study for the first time.

## 2 Material and methods

### 2.1 Data collection and expression profiling of non-cellular secretory components

The data for 306 non-cellular secretory components, including chemokines, cytokines, and growth factors, were extracted from PubMed, Google Scholar, and Scopus. Chemokines, cytokines, and growth factors were expressed differently in GBM patients when compared to normal tissue utilizing several web servers that included GBM patients’ transcriptomics data such as RNA sequencing data [(Gene Expression Profiling Interactive Analysis (GEPIA2.0, http://gepia.cancer-pku.cn/index.html), UCSC Xena **R2Q6** (https://xena.ucsc.edu/), GlioVis-TCGA(http://gliovis.bioinfo.cnio.es/)] and microarray data [GlioVis-REMBRANDT, GlioVis-AGILENT, GlioVis-HG-U133, and GlioVis-GRAVENDEEL] and proteomics data such as Osppc (https://bioinfo.henu.edu.cn/Protein/OSppc.html) (Gravendeel et al., 2009; Madhavan et al., 2009; Bowman et al., 2017; Tang et al., 2019; Goldman et al., 2020; OSppc, 2022). GEPIA2.0 and UCSC XENA compare TCGA and GDC tumor samples with matched Genotype-Tissue Expression (GTEx) standard samples. Venn analysis was performed using Venny2.1 (https://bioinfogp.cnb.csic.es/tools/venny/) to identify common DEGs from transcriptomics (RNA sequences and microarray) and proteomics data (CPTAC).

### 2.2 Gene-set enrichment and pathway analysis of differentially regulated proteomics signatures

Functional enrichment analysis of the Kyoto Encyclopaedia of Genes (KEGG) pathways and gene ontologies (GOs) of candidate DEGs were determined through a FunRich tool (version 3.1.3) (http://www.funrich.org/) (Pathan et al., 2015) and Enrichr server (https://amp.pharm.mssm.edu/Enrichr) (Chen et al., 2013; Kuleshov et al., 2016). These tools identify and prioritize the essential genes related to GBM, followed by exploring biological pathways linked with them. A *p*-value ≤0.05 was deemed significant for GO analysis and route analysis statistical evaluation, and the fold-enrichment value was considered.

### 2.3 Analysis of prognostic relevance of identified signatures and their subcellular localization

To assess the prognostic relevance of DEGs, we performed Kaplan-Meier (KM) plots to examine the overall survival (OS) and disease-free survival (DFS) of the GBM cohorts through web servers such as GEPIA2.0 and OSgbm (http://bioinfo.henu.edu.cn/GBM/GBMList.jsp.) (Dong et al., 2020). OSgbm web server includes 684 samples with transcriptome profiles and clinical information from TCGA, Gene Expression Omnibus (GEO), and Chinese Glioma Genome Atlas (CGGA). We used the median expression as the expression threshold to divide patient samples into high- and low-expression groups for survival analyses of differentially expressed genes between GBM cohorts, along with the hazard ratio (HR), 95% confidence interval (CI), and log-rank test *p*-value. The Cox proportional hazard regression model calculated all HRs based on a high vs. low comparison. In addition, CELLO v.2.5: subCELlular LOcalization predictor (http://cello.life.nctu.edu.tw/) was used for predicting subcellular localization of biomarkers.

### 2.4 Identification of potential E2 conjugating enzyme, E3 Ligase, and substrate in GBM

E2s data was assembled through the Ubiquitin and Ubiquitin-like Conjugation Database (UUCD) (http://uucd.biocuckoo.org) (Gao et al., 2013). In addition, we collated human E3 ligase enzyme from four distinct sources UUCD databases, Database of Human E3 Ubiquitin Ligases (https://esbl.nhlbi.nih.gov/Databases/KSBP2/Targets/Lists/E3-ligases/), Cell Signaling Incorporated Database (http://www.cellsignal.com/common/content/content.jsp?id=science-tables-ubiquitin), and UbiNet 2.0 (https://awi.cuhk.edu.cn/∼ubinet/index.php) (Li et al., 2021) database. Moreover, to identify substrate associated with E3 ligase, we have explored STRING (https://string-db.org/) (Szklarczyk et al., 2021) webtool to perform protein-protein interactions based on experimental data and >0.400 confidence score, UbiNeT2.0 and Ubibrowser 2.0 (http://ubibrowser.ncpsb.org.cn) (Wang et al., 2022).

### 2.5 Correlation study between a substrate, E2 conjugating enzyme, and E3 ligase

Spearman’s correlation coefficient approach was used to investigate the correlation between two proteins in GBM samples using two web tools, GEPIA2.0 and TIMER2.0 (http://timer.cistrome.org/) (Li et al., 2020). GEPIA2.0 provides pair-wise gene correlation analysis of a given set of TCGA and/or GTEx expression data. In addition, TIMER2.0 Modules examine associations between gene expression and tumor features in TCGA. We have also performed a purity adjustment. We have studied the correlation between a) biomarker substrate with E3 ligase, and b) E2s with E3 ligase and HAT enzymes. Proteins with significant positive correlation were selected for further studies.

### 2.6 Prediction of Lysine signature for acetylation and associated HATs enzymes

Two PTM prediction webservers based on deep learning methods, such as Deep-PLA (http://deeppla.cancerbio.info) (Yu K. et al., 2020) and GPS-PAIL 2.0 (http://pail.biocuckoo.org/) (Deng et al., 2016), were used to predict acetylation sites on internal lysine residues along with seven HATs enzymes, including CREBBP, EP300, HAT1, KAT2A, KAT2B, KAT5 and KAT8. The technique predicts acetylation sites based on the idea that various HATs have unique sequence specificities for the substrate changes. GPS-PAIL trains a Group-Based Prediction System previously developed method to create a computational model for each HAT enzyme.

### 2.7 Structural analysis of selected E2 conjugating enzyme

#### 2.7.1 Prediction of secondary structure

PTM affects the secondary structure of the protein, which governs its biological functions. PSIPRED: protein structure analysis workbench (http://bioinf.cs.ucl.ac.uk/psipred/) (Buchan and Jones, 2019) was used to predict the structural selectivity of lysine acetylation sites. Subsequently, the relationship between the protein’s secondary structure, fold recognition, and its corresponding acetylating sites was established. The output result was classified into three categories such as coiled, helix, and strand.

#### 2.7.2 Protein intrinsic disorder prediction

The FASTA sequence of the protein was procured from the Uniport (https://www.uniprot.org/) (Bateman et al., 2017) database. DISOPRED3 (http://bioinf.cs.ucl.ac.uk/disopred) predicts structural order and disorder regions along with protein binding sites within disordered regions using a SVM that examines patterns of evolutionary sequence conservation, positional information, and amino acid composition of putative disordered regions. As analyzed from the output, the extracted data were separated into two categories: ordered and disordered regions.

### 2.8 Mutational analysis of Lysine modification

The functional impact of lysine mutations was investigated with the use of web applications such as PMut (http://mmb.irbbarcelona.org/PMut/) (López-Ferrando et al., 2017), SNAP2 (https://rostlab.org/services/snap/) (Hecht et al., 2015), Polymorphism Phenotyping v2 (PolyPhen2) (http://genetics.bwh.harvard.edu/pph2/) (Adzhubei et al., 2010), and MutPred2 (http://mutpred.mutdb.org/index.html) (Pejaver et al., 2020). All these tools require protein sequences in the FASTA format and a list of amino acid substitutions. The output results were computed numerically, and the combined score of the four web tools was determined. If a mutation’s confidence score is ≥2.5, referred to as a threshold value, the mutation is considered disease sensitive. The basic, charged lysine (K) residue was changed into glutamine (Q), leucine (L), glutamate (E), and arginine (R). Additionally, the software MutPred2 was employed to forecast the physical impact of a lysine mutation on acetylation. The impacted sites were divided into two groups based on whether neighbouring sites gained or lost functionality.

### 2.9 Characterization of Therapeutic axis

#### 2.9.1 ROC plotter: predictive marker identification

ROC plotter-an online ROC analysis tool (https://www.rocplot.org/) (Menyhárt et al., 2021), was employed to comprehend the association between gene expression and therapeutic response using transcriptomic level data from TCGA datasets of GBM and other cancer. This tool uses a JetSet probe to select the optimal microarray probe representing a gene. The package ‘ROC’ was used to calculate the area under the curve (AUC). The integrated database comprises 454 GBM patients from 3 independent datasets and 10103 genes. Patients were categorized as responders/non-responders based on their survival status at 16 months post-surgery.

#### 2.9.2 Expression response to top mutated gene in GBM

Literature was used to find the top 10 mutated genes in GBM. “Gene_Mutation” module of TIMER2.0 was used to compare the differential gene expression with different mutation statuses of top mutated genes (such as PTEN, TP53, EGFR, PIK3R1, PIK3CA, NF1, RB1, IDH1, PTPRD, and ERBB2) of GBM.

#### 2.9.3 Correlation with protein kinase protein GBM

KinMap, (http://www.kinhub.org/kinmap/), a user-friendly web interface for the human genome (the “kinome”) was explored to retrieve 536 human protein kinases including eight typical groups (AGC, CAMK, CK1, CMGC, STE, TK, TKL, Other) and 13 atypical families (Eid et al., 2017). Using the GEPIA2.0 tool, the expression of each kinase was examined in GBM patient tumor samples. Network analysis was employed to study the correlation between the putative ‘therapeutic axis’ proteins and significantly dysregulated kinases.

### 2.10 Statistically analysis

In GEPIA2.0, we used the ANOVA statistical method for differential gene expression analysis, selected log_2_ (TPM +1) transformed expression data for plotting, TCGA tumor compared to TCGA normal and GTEx normal for matched normal data in plotting, |log_2_FC| cut-off of 1.5, and a q-value cut-off of 0.05. For survival analysis, it uses the Mantel-Cox test for the hypothesis test. OSppc used Mann-Whitney Wilcoxon tests to calculate the significant difference between proteomics data of tumors and adjacent normal tissues. In the TIMER2.0 database analysis, partial Spearman’s correlation (ρ) was applied. When Rho, *ρ* > 0.1, it indicated a correlation between the genes and immune cells. Red color signifies: Positive correlation (*p*-value <0.05, *ρ* > 0), blue color signifies: Negative correlation (*p*-value<0.05, *ρ* > 0), and grey color signify: non-significant (*p*-value >0.05).

## 3 Results and discussion

### 3.1 Expression of secretory components in GBM and normal tissue

**FIGURE 2:**
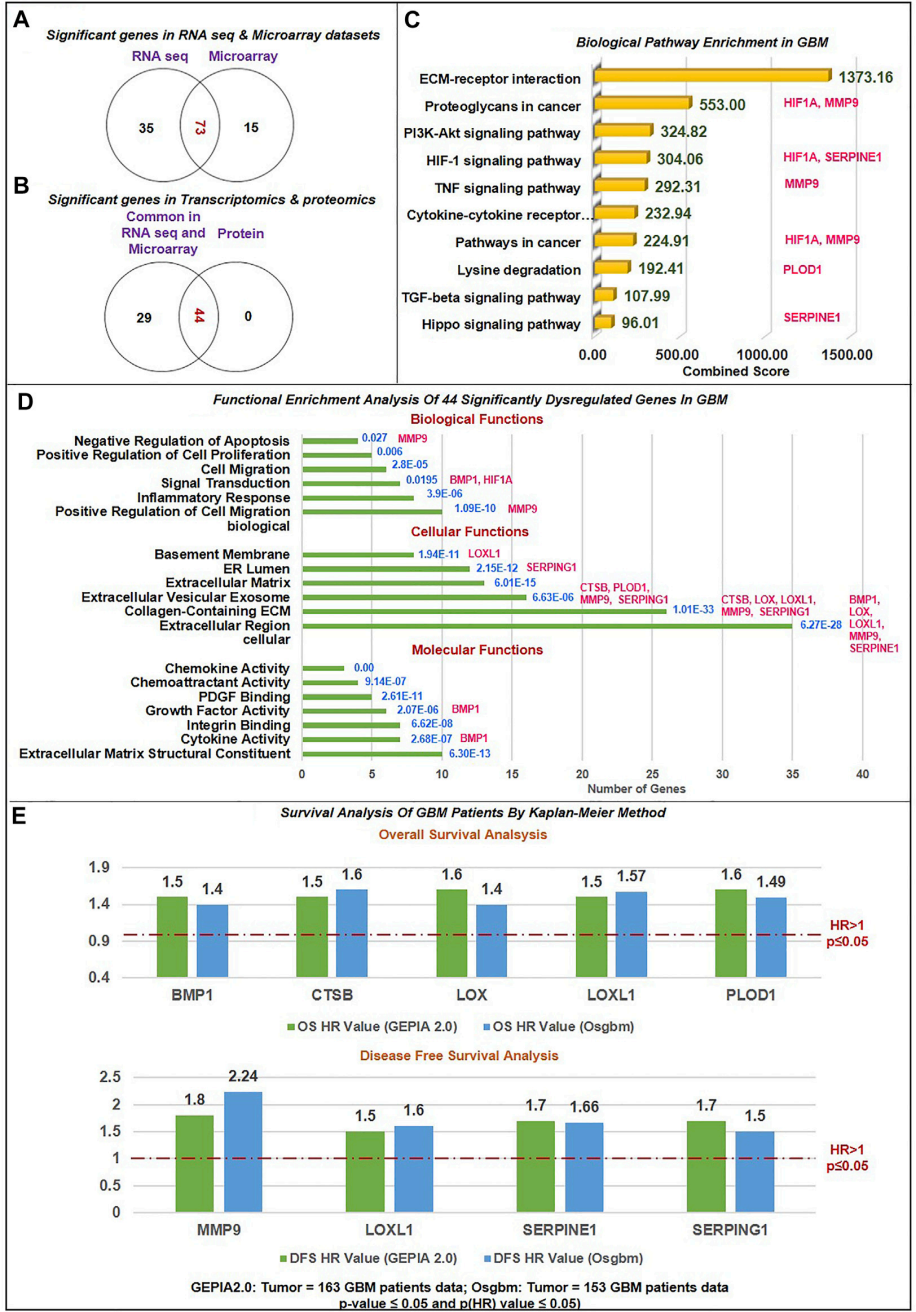
Data sorting and functional enrichment of significant non-cellular secretory biomarkers: **(A)** Venn diagram showing significant differentially expressed genes from transcriptomics data (RNAseq and Microarray) datasets. 73 genes overlap in RNA and microarray datasets **(B)** Venn diagram showing significant differentially expressed genes common in transcriptomics and proteomics datasets of GBM with the cut-off criteria of |log_2_FC| ≥ 1.5 and *p*-value≤ 0.05. 44 genes are common with protein datasets. **(C)** Biological pathway analysis using KEGG pathway: Among the top 10 biological pathways based on combined score* (written in green color) calculated by Enrichr tool are ECM-receptor, P13K-Akt, Hypoxia, TNF, TGF, and Hippo pathways with *p*-value≤0.05 in GBM. *Combined score is computed by taking the log of the value from the Fisher-exact test and multiplying that by the z-score of the deviation from the expected rank. Potential biomarkers identified in the current study have been mapped in front of each pathway. **(D)** Gene ontology (GO) analysis contains three sub-ontologies: molecular function, cellular components, and biological process associated with 44 biomarkers. Molecular function and cellular components showed maximum numbers of biomarkers involved in ECM structural constitute and localized extracellular region. At the same time, top-ranked biological processes are extracellular matrix organization, cell migration, inflammation, response to hypoxia, signal transduction, and angiogenesis. Blue text showing the *p*-value of this analysis. Potential biomarkers identified in the current study have been mapped in front of each bar of the graph. **(E)** Survival Analysis of GBM Patients by Kaplan-Meier Method: The Cox proportional Hazard ratio (HR) was plotted against prognostic markers. GEPIA and Osgbm perform overall survival (OS) or disease-free survival (DFS) analysis based on gene expression. It uses the Log-rank test and the Mantel-Cox test for the hypothesis test. Threshold HR value > 1 signifies poor prognostic markers, and HR < 1 represents good prognostic markers. Based on OS analysis over expression of BMP1, CTSB, LOX, LOXL1and PLOD1 and DFS overexpression of MMP9, LOXL1, SERPINE1, and SERPING1 were significantly associated with poor prognosis in GBM. *Green bar color*: Data from GEPIA2.0 webtool; *Blue bar color*: Data from Osgbm webtool.

The 306 non-cellular secretory components, including chemokines, cytokines, and growth-factor of TME, have been extracted from PubMed and Google Scholar. A total of 53 chemokines, including all 4 subfamilies CXC, CC, CX3C, and C (Gao et al., 2022), 253 cytokines and growth-factors including ILs, IFNs family, TNFs family, TGFs superfamily (BMP-like family, GDNFs family, TGF-β-like family), MMPs family, FGFs family, PDGFs family, VEGFs, TIMPs, prolactin, GCSFs, GMCSFs, were extracted. Firstly, we have studied the expression of chemokines, cytokines, and growth factors in GBM at transcriptomics and proteomics levels using a web tool based on TCGA data sets. RNA sequence data were analyzed using GEPIA2.0 (163 GBM tissue and 207 normal tissue, including GTEx normal tissue), UCSC Xena (154 GBM tissues and 5 Normal tissues), GlioVis-TCGA (156 GBM tissues and 4 Normal tissues), and microarray data were analyzed using GlioVis-REMBRANDT (225 GBM tissues and 28 Normal tissues), GlioVis-AGILENT (489 GBM and 10 normal tissues), GlioVis-HG-U133 (528 GBM tissues and 10 normal tissues), and GlioVis-GRAVENDEEL (117 GBM tissues and 8 normal tissues), and protein data from CPTAC, RPPA, and TCGA were analyzed using Osppc tool. We have used the Venny2.1.0 database to identify all non-cellular secreted components of TME that were significantly expressed in at least four RNA sequence data and microarray data. 73 genes were commonly expressed in RNA sequence and microarray data (Figure 2A). Afterward, the protein expression of these 73 genes was checked. A total of 44 biomarkers has significantly dysregulated expression (log_2_FC score ≥1.5 and *p*-value ≤0.05), out of which 41 were upregulated and 3 downregulated in patients with GBM compared with its normal tissues (Figure 2B). Thus, the details expression pattern of 306 secretory components has been tabulated in Supplementary Information Supplementary Table S1, and 44 shortlisted biomarkers were tabulated in Table 1 (Description in Supplementary Table S2. Previous studies also support our observations. Out of 44, only 3 were chemokines in which CCL5 and CXCL16 were upregulated, whereas CX3CL1 was downregulated in GBM. A study by Dai et al., 2016 showed that CCL5 chemokines influence tumor progression through various mechanisms that directly affect cancer cell proliferation or indirectly regulate angiogenesis and recruitment of immune cells that promote tumor growth and metastasis (Dai et al., 2016; Takacs et al., 2021). In addition to tumors, tumor-associated cells such as CAF, EC, MSC, MDSC, and TAM generate CXCL16 and influence tumor-associated cells in glial tumors (Hattermann et al., 2013; Korbecki et al., 2021). Cytokines and growth factors have a pleiotropic role in influencing various biological functions, including immune response, inflammation, and cell-to-cell communication. Studies on GBM provide evidence to support our observation of cytokines. For instance, Frei et al., 2015 demonstrated that TGFβ acts as a critical molecule implicated in GBM malignancy (Frei et al., 2015). Other studies show the importance of IL-18 in cell migration, which is fatal and untreatable, and the mechanism through which GBM cells release ECM proteins like fibronectin and vitronectin, in turn, causes the surrounding normal brain microglia to secrete more IL-18 (Yeh et al., 2012; Kast, 2015).

**TABLE 1:** Transcriptomics and proteomics expression analysis of non-cellular secretary components in GBM patients samples compared with normal tissues.

Webtools		RNA sequence datasets						Microarray datasets										Protein expression		Molecular function
		*GEPIA 2.0*		*UCSC XEna*		*GLIOVIS*		*GLIOVIS*						*TCGA_GBM*				*CPTAC*		
		TCGA GBM_GTX		TCGA GBM	GDC TCGA GBA	TCGA RNA sequence		REMBRANDT			GRAVENDEEL			HG-U133A		AGILENT-4502A		Osppc		
Chemokine	CCL5																			The CCL5/CCR5 axis regulates the infiltration, and interactions with, mesenchymal stem cells, which constitute niches
	CX3CL1																			encourage pro-tumorigenic effects, angiogenesis
	CXCL16																			employs the CXCR6 receptor to trigger glial progenitor cells to migrate and invade
Cytokines and Growth factors	ANGPT2																			a Tie2 antagonistic ligand has been linked with a poor outcome in GBM patients
	BMP1																			oncogenic role and is implicated in the invasion of GBM cells
	BMP7																			enhance transmigration, migration, and invasion of GBM cells
	COL1A1																			important ECM component that encourages invasion and tumor growth
	COL1A2																			increase GBM cell invasion and proliferation
	COL3A1																			promotes EMT and immune infiltration
	COL4A1																			boosted cancer-related pathways, including cell cycle control and the JAK/STAT signaling pathway
	COL4A2																			correlates with immune cell infiltration
	COL5A1																			enhances tumor immune tolerance, which has a negative prognosis
	COL5A2																			The outcome of LGG is negatively impacted by COL5A2 overexpression
	CTSB																			immunosuppression, immune cell infiltration, and poor prognostic indicators
	HIF1A																			Under high HIF1A expression, T-cell exhaustion-related gene expression levels and immune cell numbers increased
	IL-18																			IL-18 produced by microglia causes GBM cell movement and encourages centrifugal migration
	LAMA4																			GBM selectively secreted protein in CSF
	LAMA5																			stimulates VEGF activity, which reduces invasion but promotes tumor development by increasing GBM cell adhesion to blood arteries
	LAMB1																			The ERK/c-Jun Axis-Mediated Upregulation of LAMB1 Enables Gastric Cancer Progression and Motility
	LGALS3																			relates to tumor risk and prognosis and results in treatment resistance
	LGALS9																			Exosomal LGALS9 from GBM cells controls the growth of tumors by preventing the presentation of DC antigens and the activation of cytotoxic T cells
	LOX																			regulates the expression of MMP2,9 and is involved in the proliferation
	LOXL1																			interact with several antiapoptosis modulators (BAG2) to display antiapoptotic action
	LOXL3																			associated with genomic stability, cell proliferation, and metastasis in GBM
	MMP14																			involved in radiosensitivity, cell migration, and invasion
	MMP17																			tumorigenesis
	MMP2																			degradation of IV collagen, an important marker in glioma genesis
	MMP9																			by virtue of their proteolytic action, degrades gelatin, collagens IV, and V in the ECM
	PLOD1																			Promotes tumor via HSF1 signaling pathway
	PLOD2																			influences both tumor progression and the immune microenvironment
	PLOD3																			promotes tumor progression and poor prognosis
	PTGES2																			not much studied in GBM. In breast cancer: high expression has an immunomodulatory role
	SDF2																			overexpressed in breast cancer
	SDF4																			overexpresses in pancreatic cancer
	SERPINE1																			Influence cell-substrate adhesion and directional movement of GBM cells through TGFβ signaling
	SERPING1																			produced primarily by monocytes and works by blocking the traditional complement system pathway
	SPP1																			high SPP1 expression promotes the GSCs properties and radiation resistance and is correlated with poor prognosis of GBM
	TGFβ1																			modulates temozolomide resistance in GBM
	TGFβ2																			promote EMT
	TIMP1																			transcriptional factor Sp1 binds to the promoter of TIMP1 and triggers its expression and immune infiltration in GBM.
	TIMP3																			high TIMP3 expression correlated with better overall survival (OS) and disease-specific survival (DSS) in GBM patient
	TNFAIP6																			promotes invasion and metastasis
	TNFAIP6																			promotes invasion and metastasis
	VEGFA																			GSCs secrete the pro-angiogenic VEGF-A factor in extracellular vesicles
Patient samples number used in the respective study																				
TUMOR			**163**	**154**	**155**		**156**		**225**			**117**			**528**		**489**		**153**	
N0N-TUMOR			**207**	**5**	**5**		**4**		**28**			**8**			**10**		**10**		**__**	
Upregulated in GBM							p ≤ 0.001						p ≤ 0.001				p ≤ 0.05			
Downregulated in GBM							p ≤ 0.001						p ≤ 0.001				p ≤ 0.05			
Not significant in GBM					**p > 0.05**															

A comprehensive investigation of TIMPs in GBM by Han et al. revealed that TIMP3 indirectly controls MMPs signaling and ECM remodeling (Han et al., 2021). Multiple hormonal and non-hormonal growth-stimulating agents are also present in GBM and can function as biomarkers (Dahlberg et al., 2022). Recent research has also emphasized the critical role played by these secretory components in the pathogenesis of GBM and the creation of the immune milieu through immunological regulation, which inhibits anti-tumor responses and promotes the growth of tumors (Yeo et al., 2021). Thus, our results further confirm these previous findings.

### 3.2 Functional enrichment and biological pathway analysis of biomarkers

We have performed functional enrichment analysis using the FunRich-functional enrichment analysis tool for (GO) and KEGG pathway enrichment analysis to investigate the role of 44 differential biomarkers in GBM. We selected only pathways that were involved in the pathogenesis of the GBM microenvironment and had a large number of genes with significant fold enrichment. We have also looked at how biomarkers are involved in the biological processes that lead to the pathology of GBM. According to the results of cellular components, the bulk of biomarkers is located in extracellular regions, the ECM, and extracellular vesicles (EVs). These data corroborate earlier findings that secretory components, which are located in the extracellular space of the microenvironment and have a variety of clinical implications, have the ability to function as biomarkers and potentially disrupt signaling pathways implicated in tumorigenesis (Liu C. et al., 2021). Cytokines are soluble factors released predominantly in soluble or EV-associated forms and are involved in cell-cell communications (Fitzgerald et al., 2018). Molecular function analysis showed that the maximum number of biomarkers were engaged in structural components of ECM, cytokines and chemoattractant activities, integrin binding, growth-factors activities, and Platelet-derived growth factor binding. Chemokines act as chemoattraction, which binds to G protein-coupled seven transmembrane cell surface receptors (GPCRs) and thus activates a cascade of signaling G proteins, PI3K, protein kinase C, phospholipase C, RAS, and MAPKs to mediate immune cells migration, activation, cell chemotaxis, invasion, production of mediators promoting angiogenesis, and transactivation of EGFR (Zhou J. et al., 2014). Studies showed that the expression of specific integrins is upregulated in both tumor cells and stromal cells in a TME. Integrins receptors bind to specific secretory components from TME, which regulate ECM detachment, migration, invasion, proliferation, and survival through PI3K-AKT signaling (Ellert-Miklaszewska et al., 2020).

Biological process analysis showed top six processes were ECM organization, cell migration, inflammatory response, response to hypoxia, and angiogenesis. Additionally, we used the Enrichr tool to examine the KEGG Pathway 2021. We studied the biological pathway causing the pathology of GBM. According to the tool’s combined score, the top 10 biological pathways were ECM-receptor interaction, proteoglycans in cancer, PI3K-Akt signaling pathway, HIF1 signaling pathway, TNF signaling pathway, cytokine-cytokine receptor interaction, lysine degradation, TGF-β signaling pathway, and Hippo signaling pathway. Previous studies have found that activation of the HIF1A pathway is a common feature of gliomas and may explain the intense vascular hyperplasia often seen in GBM (Kaur et al., 2005; Domènech et al., 2021).

Similarly, TNF signaling enhances invasion in GBM and upregulates MEK-ERK signaling, NF-κB1, and STAT expression (Ramaswamy et al., 2019). In GBM, TNF secreted by the associated macrophages with the tumor encourages the activation of endothelial cells, which makes the patient resistant to anti-angiogenic treatments (Wei et al., 2021). Similar to increased PI3K-AKT activation, it has a distinct function in tumor growth but does not cause resistance to treatment (Langhans et al., 2017). There is mounting evidence that Hippo signaling has a role in a number of cancers, including glioma, breast, lung, and colon cancer. The concept that this route might represent a potential target opening the door for alternative medicines is supported by the fact that it is less studied in GBM and engaged in tumorigenesis and metastasis (Masliantsev et al., 2021). Our pathways analysis results also line up with previous findings (Ellert-Miklaszewska et al., 2020). Herein, through the top-mentioned molecular functions and biological pathways, we have demonstrated that the majority of the shortlisted secretory biomarkers were localized in extracellular space and were critical for tumorigenesis, migration, and invasion in the pathology of GBM. As a result, these signaling pathways have the potential to be further investigated in the context of GBM development and can be therapeutically addressed if we intend to target the GBM microenvironment in addition to the tumor cells. Figures 2C,D demonstrate all biological pathways and GO analysis of 44 biomarkers, respectively.

### 3.3 Relationship between biomarkers and survivals of GBM patients

To evaluate the relation between 44 significantly differentially expressed genes and the prognosis of GBM patients, GEPIA2.0 and OSgbm web tools were used for plotting KM plots for OS and DFS analysis. These tools use GBM data from TCGA. The data was analyzed in KM plot where curves were stratified by median signal expression (high vs. low expression group). The cox proportional HR and *p*-values are displayed on survival curves. A *p*-value ≤0.05 was considered statistically significant, HR > 1 was considered a poor prognostic, and HR < 1 was a good prognosis. Figure 2E and Supplementary Figure S1 illustrate the strong association of overexpression of bone morphogenetic *protein* 1 (BMP1), cathepsin B (CTSB), lysyl oxidase (LOX), procollagen-lysine,2-oxoglutarate 5-dioxygenase 1 (PLOD1) with poor OS (HR > 1 and p (HR) ≤ 0.05). CTSB proteases are essential in ECM degradation and are overexpressed in most human colon and other cancers. A recent study by Ma et al. (2022) also demonstrates that CTSB is a negative prognostic biomarker and biological pathway associated with immune suppression and inflammation in glioma (Ma et al., 2022). Studies have demonstrated that CTSB regulates several forms of cell death, such as apoptosis, necroptosis, autophagy, pyroptosis, and ferroptosis, and is associated with radio-resistance, tissue invasion, and metastasis of GBM (Ding et al., 2022)**.** BMP1 (secreted metalloprotease of the astacin metalloproteinase family) recently emerged as a cancer-related protein in multiple cancer but is less explored in GBM. Signaling such as TGFβ involving BMP1 affects the proliferation and differentiation of glioma stem cells. According to the study by Xiao et al. (2019), increased expression of BMP1 reflects poor prognosis in clear cell renal cell carcinoma (Xiao et al., 2019). Similarly, we first time reported that BMP1 had poor OS in GBM patient samples. A study by Sachdeva et al., in 2019 showed that in the GBM microenvironment dysregulated BMP signaling via expression of p21 protein causes GSCs to enter a quiescent state, rather than developed into the differentiated astroglia cell (Sachdeva et al., 2019). In addition, a study showed that increased expression of LOX expression was strongly associated with the invasive features of malignant astrocytes. LOX is well recognized as secreted matrix-modifying enzyme. The key roles played by LOX include the regulation of gene expression, protein-lysine 6-oxidase activity, protein binding, and protein phosphorylation. It has an impact on cell cycle progression and apoptosis in GBM and can be exploited as a target for early detection and targeted treatment (Zhang P. et al., 2022; Zhang S.et al., 2022). Li et al. (2021) showed that ECM-related gene LOX correlated with poor OS in glioma patients (Li et al., 2022), including GBM (Tang et al., 2020) and gastric cancer (Zhu et al., 2021). Another investigation discovered a difference between Lysine oxidase-like 1 (LOXL1) and poor OS in GBM (Liu Z. et al., 2021). The antiapoptotic activity of LOXL1 is mediated via interactions with a variety of antiapoptotic modulators, including BAG2, and by Wnt/beta-catenin signaling (Yu H. et al., 2020). Our finding revealed that the upregulation of LOXL1 was accompanied by both poor OS and DFS. Moreover, PLOD1 encourages cross-linking in ECM molecules, enabling ECM structural stability and maturation. In a study by Wang et al. (2020), increased PLOD1 expression in glioma was linked with a worse prognosis (Wang et al., 2020). Significant overexpression of PLOD1 may encourage the growth and colony formation of U87 cells by triggering the HSF1 signaling pathway (Yuan et al., 2022) however, in hypoxic settings could stimulate invasiveness and the mesenchymal transition by inducing NF-κB signaling pathway (Wang et al., 2021). Secondly, our data demonstrated the overexpression of Matrix metallopeptidase 9 (MMP9), Serpin Family E Member 1 (SERPINE1), and serine protease inhibitor family G1 (SERPING1) linked with poor DFS (HR > 1 and p (HR)≤0.05) (Figure 2E and Supplementary Figure S2A). Our finding supported previous studies that the overexpression of MMP9 indicates a poor prognosis in glioma (Zhou et al., 2019). In the microenvironment GBM-secreted factors influence increased human brain vascular endothelial cell migration as well as levels of MMP-9 and CXCR4 which result in enhanced angiogenesis (De Oliveira Rosario et al., 2020). Indeed, Seker et al. (2019) research shows that poor patient survival in GBM is related to increased expression of SERPINE1 (Seker et al., n. d.). In hypoxic microenvironment condition, ROS promotes tumor progression, EMT in GBM through HIF1A-SERPINE1 signaling (Zhang et al., 2023). In another study, it was found out that low SERPING1 levels have been associated with poor DFS in prostate cancer (Peng et al., 2018) In contrast, our study reported a higher level of SERPING1 linked with poor DFS/prognosis in GBM. These results showed that BMP1, CTSB, LOX, LOXL1, MMP9, SERPINE1, and SERPING1 are poor prognostic indicators in GBM since they had HR > 1 and p (HR) ≤ 0.05. Jia et al. (2018) also showed that SERPINE1 and SERPING1link with poor prognosis in GBM (Jia et al., 2018).

**FIGURE 3:**
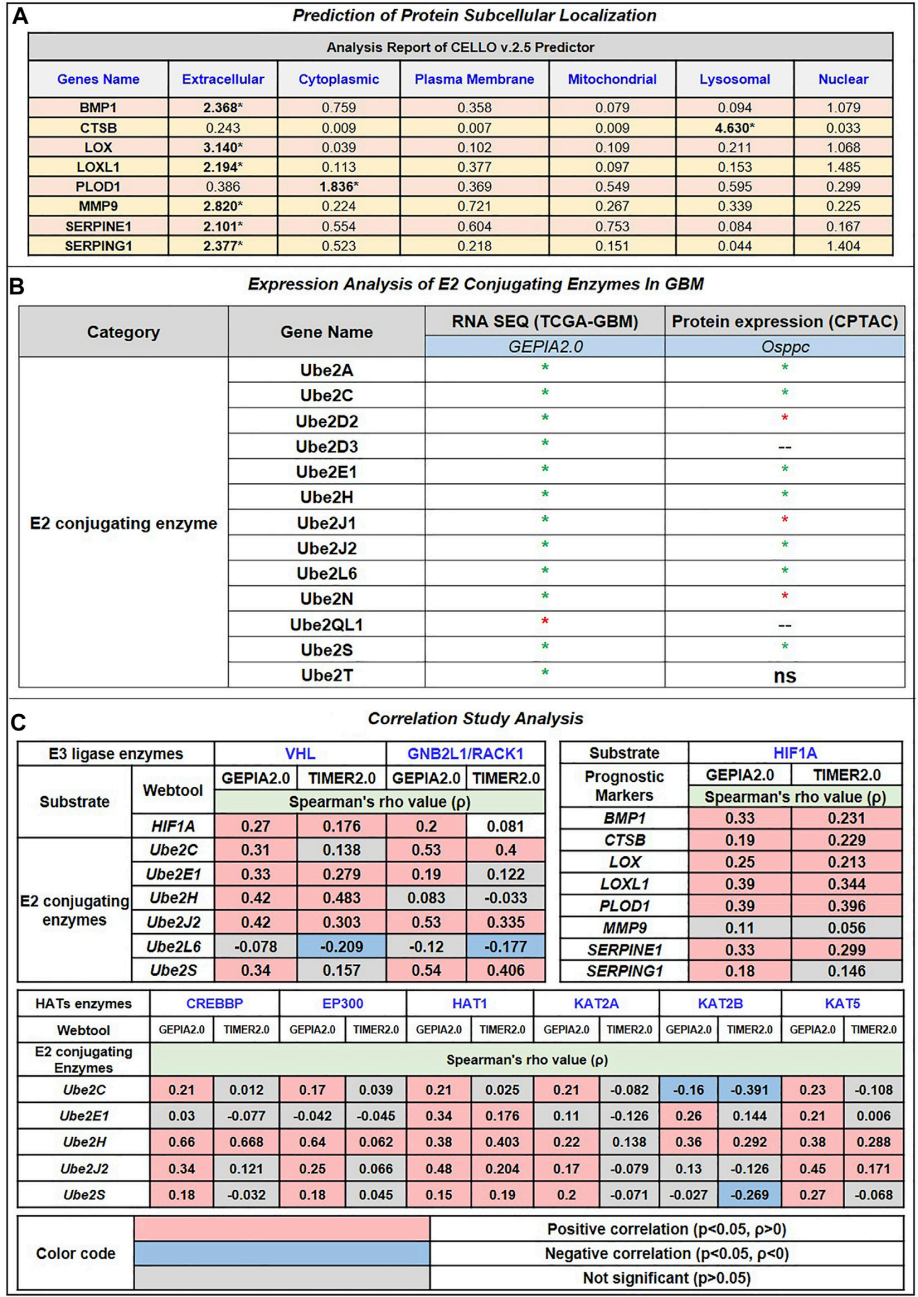
**(A)** Prediction of Protein subcellular localization by cello online predictor: BMP1, LOX, LOXL1, MMP9, SERPINE1, and SERPING1 localized in majorly extracellular space. CTSB is majorly localized in lysosomes and PLOD1 in the cytoplasm, followed by extracellular space. **(B)** Expression analysis of E2 conjugating Enzymes (E2s). Out of 35 reported E2s in humans, at the mRNA level, only 13 were dysregulated (including 12 up and 1 downregulated); at the protein level, 10 were dysregulated (including 7 upregulated and 3 downregulated). **(C)** Correlation study analysis: E3 ligase, VHL, and GNB2L1 showed a significant positive correlation with substrate HIF1A and E2s. VHL showed a significant positive correlation between Ube2E1, Ube2H, and Ube2J2, while GNB2L1 showed a positive correlation with Ube2C, Ube2J2, and Ube2S. In addition, HIF1A positively correlates with poor prognosis markers such as BMP1, CTSB, LOX, LOXL1, PLOD1, and SERPINE1. Heatmap 3 showed a significant correlation between HAT enzymes and E2s. Results showed that Ube2H positively correlates with CREBBP, EP300, HAT1, KAT2B, and KAT5. Ube2S with HAT1, Ube2J2 with HAT1 and KAT5, and Ube2C negatively correlate with KAT2B.

Moreover, we have also used CELLO v.2.5: subCELlular LOcalization predictor for finding the localization of identified prognostic markers. Results in Figure 3A showed that BMP1, LOX, LOXL1, MMP9, SERPINE1, and SERPING1 localized in extracellular space while PLOD1 localized majorly in cytoplasm followed by extracellular space and CTSB localized in lysosome followed by extracellular space. Studies have revealed a strong correlation between a protein’s subcellular location and function. Sequencing similarity is helpful in predicting subcellular localization for sequences containing >30% sequence identity.

### 3.4 Identification of HIF1A as the substrate from dysregulated biomarkers and its associated E3 ligase

To find the therapeutic axis to understand ubiquitination systems in GBM, we have focused on finding the possible substrate from the list of 44 differentially expressed biomarkers. We have used the STRING database to find the experimentally validated (confidence score>0.400) substrate and correspondence E3 ligase. The E3 ligase list was created by combining E3 ligase protein from four different sources: the Human E3 ligase database, CST, UUCD, and UbiNet 2.0. This list was used to make an individual PPI network with every 44 biomarkers in the STRING database. This study’s results showed that BMP1, HIF1A, and TNFRSF1B are the biomarkers that also act as a substrate for E3 ligase and are involved in the Ubiquitination pathway. Results showed E3 ligase correspondence to substrate a) BMP1 was RMND5A, b) HIF1A were EP300, GNB2L1, MDM2, PARK2, STUB1, TRAF6, VHL, FBXW7, SIAH1, SIAH2, c) TNFRSF1B were TRAF1, TRAF2, ASB3, SMURF2. Subsequently, mRNA and protein expression of these substrate and their corresponding E3 ligases were studied in GBM patients (Table 2). Based on the results, only substrate HIF1A and its E3 ligase von Hippel-Lindau (VHL) and GNB2L1 were dysregulated in GBM patients’ samples both at transcriptomics and proteomics levels. Under the normoxic condition, HIF1A is ubiquitinated by VHL and E3 ligase for proteasome degradation in the cytoplasm. Once stabilized, HIF1A translocate to the nucleus, guided by a nuclear localization signal in its C-terminus (Tanimoto et al., 2000; Yu et al., 2001).

**TABLE 2:** Expression analysis of substrate and its associated E3 ligase in GBM patients samples.

Substrate (STRING, Ubibrowser2.0, Ubinet2.0)	E3 ligase (UUCD, CST, UbiNet2.0)	Combined score (STRING)	Expression in GBM	
			Gene expression (GEPIA2.0)	Protein expression (Osppm)
***BMP1***	**RMND5A**	**0.483**		
***HIF1A***	**EP300**	**0.999**		
	**GNB2L1**	**0.998**		
	**MDM2**	**0.997**		
	**PARK2**	**0.762**		
	**STUB1**	**0.81**		
	**TRAF6**	**0.72**		
	**VHL**	**0.999**		
	**FBXW7**	**0.664**		
	**SIAH1**	**0.43**		
	**SIAH2**	**0.543**		
***TNFRSF1B***	**TRAF1**	**0.761**		
	**TRAF2**	**0.881**		
	**ASB3**	**0.485**		
	**SMURF2**	**0.57**		
**Sample size**	**Tumor tissues**		**163**	**153**
	**Normal tissues**		**207**	**--**

*Green gradient signifies: significantly overexpressed in GBM, patient’s samples (*p* < 0.05).

*Red gradient signifies: significantly downregulated in GBM, patient’s samples (*p* < 0.05).

*Combined score calculated by STRING, webtool based on experimentally determined interaction data.

In contrast, Aga et al. (2014) demonstrated that endogenous HIF1A is detectable in exosomes (Aga et al., 2014) present in the microenvironment, and studies suggest that exosomes reflect the hypoxic status of glioma cells and mediate hypoxia-dependent activation of vascular cells during tumor development (Kucharzewska et al., 2013). In addition, HIF1A initiates TNFα exosome-mediated secretion under hypoxic conditions (Yu et al., 2012). In human glioblastoma cells, Bensaad et al. showed that HIF-1α was necessary to induce Fatty Acid Binding Protein 3 (FABP3) and FABP7, leading to lipid droplet accumulations (Bensaad et al., 2014). According to reports, HIF1A is essential for the growth and development of GBM as well as for tumor cell migration, glucose absorption, angiogenesis, and chemoresistance. A plethora of research showed that hypoxia triggers glioma cells to release EVs with distinct functional proangiogenic cargo, including cytokines, growth factors, proteases, and miRNA to influence endothelial cells to promote angiogenesis, metabolic, and transcriptional signaling pathways such are the EGFR, PI3K/Akt and MAPK/ERK pathways. Hypoxia-stimulated glioma EVs promote tumor vascularization, pericyte vessel coverage, and cell proliferation, eventually reducing tumor hypoxia in the GBM microenvironment (Yekula et al., 2020). Hence, we have chosen HIF1A as substrate, VHL, and GNB2L1 (another gene name: RACK1) as an E3 ligase for further studies. Earlier investigations support our observation. Mutation in VHL genes causes renal cell carcinomas, pheochromocytomas, and cerebellar hemangioblastomas (Kim and Zschiedrich, 2018). We were interested in exploring this interaction in GBM. However, based on experimental data, our analysis also proposed GNB2L1 interacting with HIF1A. Earlier, this interaction was established in breast cancer (Zhou Z. et al., 2014). Here we will discuss this in context with GBM.

Evidence from the literature suggests that the poor prognostic biomarkers LOX, BMP1, CTSB, LOXL1, PLOD1, MMP9, SERPINE1, and SERPING1 are related to the hypoxic microenvironment. First, there was a positive correlation between BMP1 and HIF1A and the malignant grade of astrocytoma, although there was no evidence of a direct or indirect association (Xiao et al., 2019). Additionally, Xiaofei et al. (2018) demonstrated that hypoxia upregulates CTSB and HIF1A in a fashion comparable to HepG2 cells. (Xiaofei et al., 2018). In several cancer types, including breast, head and neck, prostate, colon, and renal cell carcinomas, LOX controls HIF1A. The invasive and metastatic characteristics of hypoxic cancer cells, including astrocytoma, are caused by secreted LOX (Da Silva et al., 2015). Under hypoxic conditions (<1% oxygen), LOX and LOXL1 promoted angiogenesis (Xie et al., 2017). Recently, Wang et al. (2021) discovered that Hypoxia causes the overexpression of PLOD1, which, through NF-kB signaling, leads to the malignant phenotype of GBM (Wang et al., 2021). HIF1A promotes the development of MMP9, which influences invasion in breast cancer by weakening the basement membrane and the ECM barrier. HIF1A is also implicated in the control of cell proliferation, growth factor release, and angiogenesis (Choi et al., 2011). Furthermore, hypoxia-induced overproduction of reactive oxygen species (ROS) causes cancer to upregulate the SERPINE1 protein (protein that regulates cell adhesion), which controls cell adhesion in breast cancer (Azimi et al., 2017). In contrast, HIF2A, not HIF1A, controls the expression of SERPING1, which is linked to immunological infiltrations in glioblastoma (Xiao et al., 2020). Accordingly, we can state that HIF1A is a crucial biomarker that correlates with all cancer biomarkers that indicate a poor prognosis. As a result, we go forward with HIF1A and want to investigate its potential role in the therapeutic axis for treating GBM.

### 3.5 Identification of significant E2 conjugating enzyme associated with VHL and GNB2L1 in GBM

Ubiquitin-conjugating enzymes (E2s) are the central players in the trio of enzymes responsible for the attachment of ubiquitin (Ub) to cellular proteins. It plays a more prominent role in ubiquitin signaling than a middleman. The UBC domain, a central catalytic domain in E2s, has about 150 amino acids. This domain adopts an α/β-fold typically with four α-helices and a four-stranded β-sheet. Important loop regions form part of the E3-binding site and the E2 active site. Several studies have suggested the dysregulation of E2 in multiple cancer. Understanding of E2s regulation is still emerging, and it is evident that E2s can be governed by various mechanisms (Stewart et al., 2016). Hence, we explore how E2s regulate and affect others, especially our shortlisted E3 ligases VHL and GNB2L1 and substrate HIF1A in GBM. We have extracted 36 E2s expressed in humans from previously published research.

In addition, we analyzed its expression at mRNA and protein levels in GBM patient samples with the help of the GEPIA2.0 and Osppc web applications (Figure 3B). We have found that at mRNA levels, 13 E2 conjugative enzymes were significantly (*p*-value ≤0.05, log_2_FC ≥ 1.5) dysregulated in GBM patient samples, including 11 upregulated (Ube2A, Ube2C, Ube2D2, Ube2D3, Ube2E1, Ube2H, Ube2J1, Ube2J2, Ube2L6, Ube2L6, Ube2N, Ube2S, Ube2T) and 1 downregulated (Ube2QL1). In addition, amongst 13 shortlisted enzymes, we found that protein levels of 7 were upregulated (Ube2A, Ube2C, Ube2E1, Ube2H, Ube2J1, Ube2H, Ube2J2, Ube2L6, Ube2S), 3 were downregulated (Ube2D2, Ube2J1, Ube2N), 2 were (Ube2D3, Ube2QL1) were not available in the database, and UBE2T were non-significant. Thus, based on both transcriptomics and proteomics expression data analysis, we moved further with 6 E2s named Ube2C, Ube2E1, Ube2H, Ube2J2, Ube2L6, Ube2S that were overexpressed in GBM. A study by Xiang and Yan (2022), Ube2C serves as both an oncogene and a tumor suppressor gene, and its overexpression is crucial to the development of thyroid cancer (Xiang and Yan, n. d.). Moreover, another study by Pan et al. (2021) demonstrates that Ube2D3 induces the ubiquitination of the SHP-2 protein, which in turn activates STAT3 signaling, promoting tumorigenesis and glycolysis in gliomas (Pan et al., 2021).

Further, we have also studied the correlation between E3 ligase with substrate and shortlisted E2s in GBM patient’s samples using GEPIA2.0 (GBM tumor sample size, n = 163) and TIMER2.0 (GBM tumor sample size, n = 153). We have tabulated purity-adjusted partial Spearman’s rho (ρ) value which gives the degree of their correlation in the form of a heatmap (Figure 3C). We have used spearman statistical analysis, and when |ρ| > 0.1, it indicated a correlation between the genes. Red color signifies: Positive correlation (*p*-value ≤0.05, *ρ* > 0), blue color signifies: Negative correlation (*p*-value≤0.05, *ρ* > 0), and grey color signify: non-significant (*p*-value>0.05). Results showed in GBM that both E3 ligase VHL and GNB2L1 were positively correlated with its substrate HIF1A. Moreover, VHL was positively correlated with Ube2E1, Ube2H, and Ube2J2, whereas GNB2L1 was positively correlated with Ube2C, Ube2J2, and Ube2S.

Furthermore, to investigate the PTM (e.g., acetylation) that can modify lysine basic residues (lysine and/or arginine). Acetylation affects a large number of histone and non-histone proteins. Growing evidence suggests that reversible lysine acetylation of non-histone proteins regulates mRNA stability, protein localization and degradation, and protein-protein and protein–DNA interactions. The dynamic regulation of genes governing cellular proliferation, differentiation, and death depends largely on the recruitment of HATs and histone deacetylases (HDACs) to the transcriptional machinery. Several oncogenes or tumor-suppressor genes produce many non-histone proteins specifically targeted by acetylation. These proteins have a direct role in carcinogenesis, tumor growth, and metastasis (Singh et al., 2010). Researchers have found acetylation sites on Ub molecules and showed how acetylated Ub modulates E1 enzyme (Uba1) catalytic activity. On a similar note here, we explore the potential acetylation site on lysine residues and its impact on selected E2s such as Ube2E1, Ube2H, Ube2J2, Ube2C, and Ube2S in GBM (Lacoursiere and Shaw, 2021). Moreover, these E2s have in patients with anaplastic gliomas, a greater Ube2C expression was linked to mitotic cyclin degradation and a significantly reduced OS duration (Ma et al., 2016). Additionally, Ube2S is controlled by the PTEN/Akt pathway and participates in DNA repair, particularly NHEJ-mediated DNA repair, which makes chemotherapeutic drugs more sensitive to GBM (Maksoud, 2021). In a recent study, Shin et al. found a mutation (*de novo* missense variant) that resembles a variant found in a patient with neurodevelopmental abnormalities, induces irregular Ube2h function in zebrafish embryos, and results in abnormal brain development (Shin et al., 2023). In addition, according to Lim and Joo (2020), circulating Ube2H mRNA is potentially used to diagnose and treat Alzheimer’s disease (Lim and Joo, 2020). However, Ube2H has been studied in cancer, although there is little information about it in GBM (Zuo et al., 2020).

### 3.6 Identification of potential lysine (K) residues for acetylation in E2s and prediction of associated HAT enzymes

**FIGURE 4:**
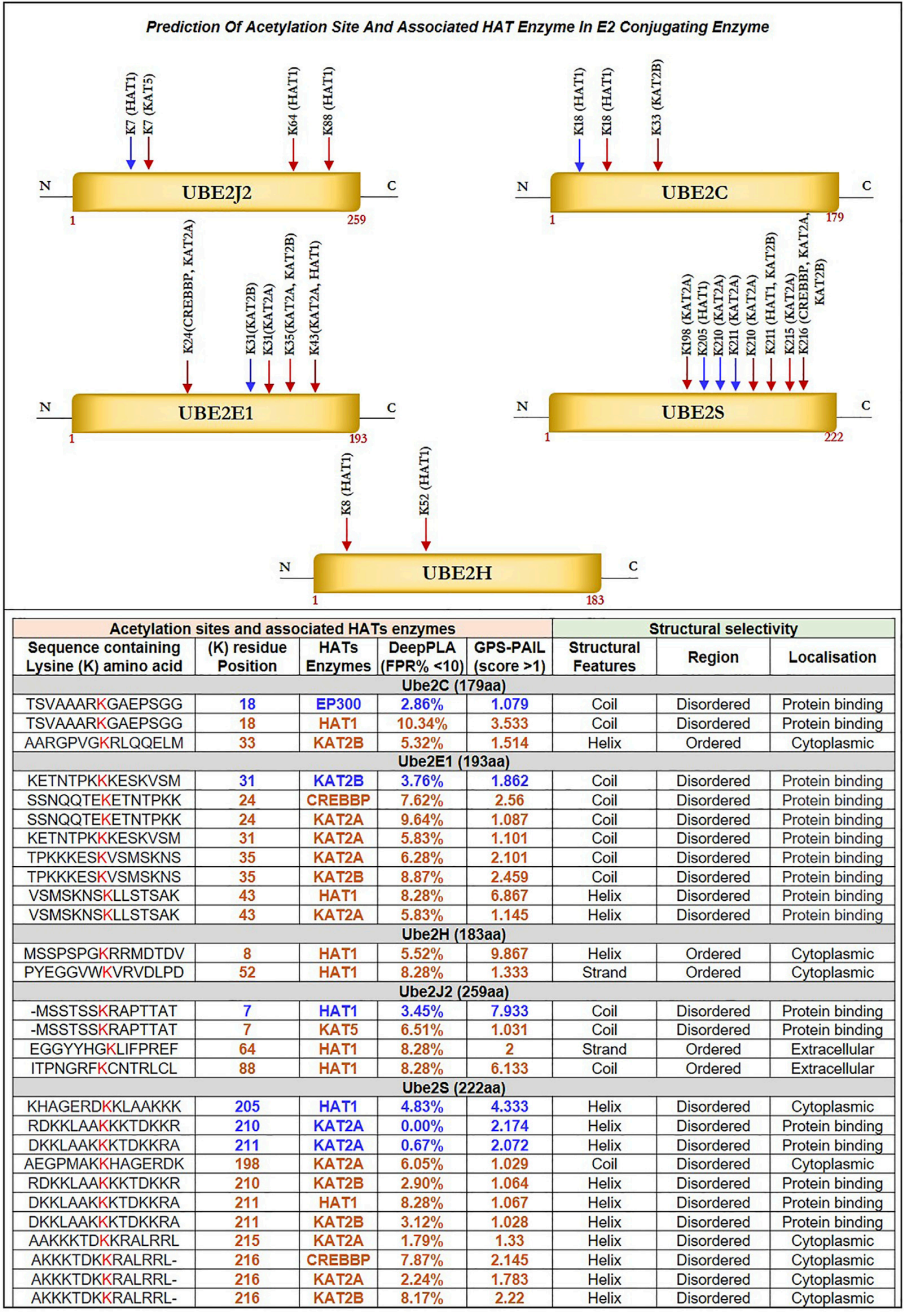
Prediction of Acetylation Site and Associated HAT Enzyme in E2 Conjugating Enzyme: Potential acetylation site on lysine residues of Ube2J2, Ube2C, Ube2E1, Ube2S, Ube2H and associated HAT enzymes were identified using DeepPLA and GPS-PAIL machine-learning based webtool. For UBE2C (K18, K33), Ube2E1(K24, K31, K35, K43), Ube2H (K8, K52), Ube2J2 (K7, K64, K88) and Ube2S (K198, K205, K210, K211, K215, K216). HAT enzymes associated with lysine residues are mentioned in the table. The lysine residue marked in blue color has a high confidence score: DeepPLA (FPR<5%) and GPS-PAIL (score>1), and the red color has a medium confidence score: DeepPLA (FPR<10%) and GPS-PAIL (score>1). In addition, structural analysis using PSIPRED and DISOPRED3 showed predicted lysine residue falls in coiled structure for Ube2C, Ube2E1, and Ube2J2 whereas, in helix structure for Ube2S. Moreover, our investigation showed acetylation occurs in disordered regions compared to ordered regions. FPR: False positive rate.

Herein, we identified acetylation sites on lysine (K) residue of shortlisted E2s such as Ube2C, Ube2E1, Ube2H, Ube2J2, Ube2S and associated HATs enzymes, including CREBBP, EP300, HAT1, KAT2A, KAT2B, KAT5, and KAT8 with using deep learning methods such as Deep-PLA and GPS-PAIL. The total ‘K’ modification sites for Ube2C, Ube2E1, Ube2H, Ube2J2, and Ube2S are 12, 15, 13, 15, and 16, respectively. We have selected only those ‘K’ residues that fall under the filter (High confidence: DeepPLA: False positive rate (FPR) % <5 and GPS-PAIL score >1; Medium confidence: DeepPLA: FPR% <10 and GPS-PAIL score >1). The extracted acetylation sites were mapped to respective proteins. Figure 4 illustrate all predicted acetylation site on ‘K’ residues and associated HATs enzymes. Our analysis observed potential acetylation ‘K’ residues that pass our filter criteria were Ube2C: K18, K33; Ube2E1 for K24, K31, K35, K43; Ube2H: K8, K52; Ube2J2: K7, K64, K88; Ube2S: K198, K205, K210, K211, K215, K216. Lacoursiere et al. (2022) have beautifully described the acetylation site in the UBC domain of 33 different E2s and its involvement in various cancer, including prostate cancer, gastric carcinoma, and leukemia. Mounting evidence from earlier studies has demonstrated acetylation sites for Ube2C (K18, leukemia), Ube2E1(K43, breast cancer), and Ube2H (K8, breast cancer) (Lacoursiere et al., 2022). Our analysis has shown novel putative acetylation sites for E2s at lysine residues are Ube2C (K33); Ube2E1 (K24, K31, K35); Ube2H (K52); Ube2J2 (K7, K64, K88); Ube2S (K198, K205, K210, K211, K215, K216).

Further, we have identified associated HAT enzymes to E2s such as for a) Ube2C: EP300, HAT1 and KAT2B; b) Ube2E1: KAT2B, CREBBP, KAT2A and HAT1; c) Ube2H: HAT1; d) Ube2J2: HAT1, KAT5; e) Ube2S: HAT1, KAT2A, KAT2B and CREBBP. These E2 can be the potential substrate for HAT enzymes. Many additional HAT substrates have been discovered in the past as a result of acetylome research, and numerous non-histone HAT substrates, including AML1, AML1-ETO (AE), p53, c-Myc, NF-κB, cohesin, and tubulin, have been identified to be crucial for a variety of cellular functions (Sun et al., 2015). Furthermore, the expression of these HAT enzymes was studied in GBM patient samples using GEPIA2.0 and OSppc tools. Analysis showed that HAT1 was upregulated while KAT2A was downregulated in GBM patient samples. Other HAT enzyme expressions, such as CREBBP, EP300, KAT2B, and KAT5, were insignificant. Hence, we moved with only upregulated HAT1 enzymes for further analysis. mRNA and protein expression data are shown in Supplementary Figure S2B.

### 3.7 Structural characterization and impact of lysine modification

Selected E2s Ube2C, Ube2E1, Ube2H, Ube2J2, and Ube2S have undergone structural characterization of the anticipated ‘K’ acetylation site as mutational investigation and its effect on disease susceptibility. Firstly, structure analysis of Ube2C, Ube2E1, Ube2H, Ube2J2, and Ube2S was performed**.** Our analysis demonstrated that Ube2E1 (3) and Ube2J2 (2) had a higher rate of acetylated ‘K’ sites falling in the coiled region, while Ube2S (6) and Ube2H (1) had a greater rate of these sites falling in helix region. Secondary structure analysis demonstrated the significance of the coiled structure in the PTM region compared to the helix and strand. Coiled areas govern protein interactions and aggregation propensity. Therefore mutations that damage coiled regions depress aggregation and protein activity, whereas mutations that improve coiled structure boost aggregation propensity (Fiumara et al., 2010).

Narasumani and Harrison (2018) demonstrated that PTMs preferred disordered regions compared to the ordered region, affecting their functions and interactions. Furthermore, the involvement of PTM in the disordered region influences disorder to order transition, thus altering the protein’s stability and associated mechanisms. In the context of eukaryotic histones, the function of acetylation has been thoroughly investigated. Acetylation of disordered tail sections stimulates gene expression by removing inhibition (Christensen et al., 2019a). However, not all PTMs prefer disordered regions (Narasumani and Harrison, 2018; Mészáros et al., 2021). Hence, we predicted the distribution of predicted acetylation in protein intrinsic ordered and disordered regions using the machine-learning-based method DISOPRED3. Results indicated that the disordered area was more likely to include possible ‘K’ acetylation residues for all five E2s, Ube2C, Ube2E1, Ube2H, Ube2J2, and Ube2S, than the ordered region. Furthermore, the localization of putative ‘K’ residue in the sequence has also been predicted; for example, the sequence containing K31 of Ube2E1 involves protein binding. Secondly, we have investigated the pathology of mutation (amino acid substitution) by substituting lysine (K) residue, which is a positively charged amino acid with each polar amino acid (glutamine, Q), non-polar (leucine, L), negatively charged (glutamate, E), and positively charged (arginine, R) through mutational analysis tools such as PMut, SNAP2, PolyPhen2 and Mutpred2. Our results observed that mutation at ‘K’ acetylation sites impacts disease susceptibility. For each tool, we have selected a score >0.5. Each numerical prediction score value has been tabulated in Table 3. However, Ube2H (K52), Ube2J2 (K64, K88) and Ube2S (K198, K210, K211, K215, K216) exhibit higher confidence scores (cumulative confidence score value > 2.5) on impact disease susceptibility. This signifies that a single amino acid substitution or mutation at identified ‘K’ residues leads to pathogenic and results in disease. Previous evidence also suggested that any mutation in these intrinsically disordered protein regions causes cancer (Mészáros et al., 2021).

**TABLE 3:** Impact of Amino Acid Substitution of “K” Putative Mutation to Either L, Q, R, Or E On Disease Susceptibility Predicted with The Help of Pmut, SNAP2, Polyphen2, and Mutpred2 tools.

Substitution	Pmut	SNAP2	PolyPhen-2	MutPred2	Total score
Ube2C					
K18L	0.74	1	0.005	0.772	2.517
K18Q	0.64	1	0.027	0.536	2.203
K18R	0.42	1	0.32	0.38	2.12
K18E	0.66	1	0.262	0.662	2.584
K33L	0.71	1	0.194	0.908	2.812
K33Q	0.59	1	0.003	0.804	2.397
K33R	0.25	1	0	0.681	1.931
K33E	0.59	1	0.049	0.868	2.507
Ube2E1					
K31L	0.49	1	0.037	0.156	1.683
K31Q	0.11	0	0.028	0.093	0.231
K31R	0.11	0	0	0.061	0.171
K31E	0.2	1	0	0.113	1.313
K24L	0.28	1	0.009	0.098	1.387
K24Q	0.09	0	0	0.066	0.156
K24R	0.09	0	0	0.044	0.134
K24E	0.11	0	0.002	0.079	0.191
K35L	0.58	1	0.09	0.196	1.866
K35Q	0.47	1	0.001	0.075	1.546
K35R	0.2	1	0	0.052	1.252
K35E	0.47	1	0.015	0.111	1.596
K43L	0.31	1	0.972	0.562	2.844
K43Q	0.2	0	0.924	0.368	1.492
K43R	0.12	1	0.007	0.211	1.338
K43E	0.35	1	0.896	0.369	2.615
Ube2H					
K8L	0.53	1	0.016	0.872	2.418
K8Q	0.51	1	0.437	0.758	2.705
K8R	0.26	1	0	0.661	1.921
K8E	0.39	1	0.354	0.831	2.575
K52L	0.63	1	0.82	0.943	3.393
K52Q	0.53	1	0.762	0.894	3.186
K52R	0.26	1	0.001	0.821	2.082
K52E	0.57	1	0.532	0.924	3.026
Ube2J2					
K7L	0.34	1	0.032	0.481	1.821
K7Q	0.37	0	0.897	0.266	1.533
K7R	0.19	0	0.868	0.205	0.395
K7E	0.33	1	0.020	0.346	1.676
K64L	0.62	1	1	0.704	3.324
K64Q	0.59	1	0.96	0.504	3.054
K64R	0.39	0	0.542	0.208	1.14
K64E	0.59	1	0.996	0.509	3.095
K88L	0.55	1	0.908	0.877	3.335
K88Q	0.48	1	0.071	0.704	2.255
K88R	0.46	1	0.009	0.538	2.007
K88E	0.52	1	0.503	0.812	2.835
Ube2S					
K198L	0.72	1	0.999	0.567	3.286
K198Q	0.45	1	0.997	0.285	2.732
K198R	0.4	1	0.996	0.186	2.582
K198E	0.44	1	0.779	0.383	2.602
K205L	0.36	1	0.133	0.529	2.022
K205Q	0.37	0	0.531	0.255	1.156
K205R	0.15	0	0.358	0.148	0.656
K205E	0.27	1	0.187	0.349	1.806
K210L	0.73	1	0.997	0.833	3.56
K210Q	0.52	1	0.999	0.559	3.078
K210R	0.29	1	0.996	0.39	2.676
K210E	0.45	1	0.996	0.686	3.132
K211L	0.68	1	0.997	0.683	3.36
K211Q	0.64	1	0.999	0.433	3.072
K211R	0.16	1	0.996	0.2	2.356
K211E	0.52	1	0.996	0.475	2.991
K215L	0.69	1	0.997	0.817	3.504
K215Q	0.7	0	0.999	0.576	2.275
K215R	0.48	0	0.996	0.365	1.841
K215E	0.79	1	0.996	0.664	3.45
K216L	0.88	1	0.997	0.859	3.736
K216Q	0.77	1	0.999	0.639	3.408
K216R	0.74	0	0.996	0.455	2.191
K216E	0.8	1	0.996	0.751	3.547

*For SNAP2 = Probable Benign: Marked as “0”; Probable damage: Marked as “1”.

*For Pmut, MutPred2, and PolyPhen-2: Effect or Probable damage = >0.5 threshold.

*Gradient of the Green color showed Total confidence score (cumulative score of Pmut, SNAP2, MutPred2, and PolyPhen-2): Higher green color signifies a high confidence score.

Subsequently, we were interested in anticipating the molecular mechanism of pathogenicity due to mutation at the ‘K’ acetylation site through the Mutpred2 web application. Supplementary Information Supplementary Table S3 demonstrates the functional impact of putative ‘K’ residue mutation on acetylation. The combined results depict the role of putative ‘K’ mutation on other cellular functions. The results revealed that mutation in Ube2C (K33), Ube2H (K8), and Ube2S (K198, K205, K210, K211, K215, and K216) results in loss of acetylation on the same site. These findings confirm what we had already noticed. Thus, loss of acetylation with a mutation at K8 for Ube2H and at K198, K205, K210, K211, K215, and K216 for Ube2S signifies our predicted lysine residue is site acetylation, and any mutation will lead to disease. Other mechanisms, along with affected motifs, have been elaborated in Supplementary Information Supplementary Table S3. Moreover, selected disease-susceptible mutations were subjected to investigate their impact on protein structure stability. Mutation at Ube2C (K18) with (E), Ube2H (K8) with (R) and Ube2S (K210, K216) with (E) and (Q) leads to the gain of helix structure. This also signifies mutation at these acetylation sites will cause a topological change in the secondary structure.

### 3.8 Prediction of therapeutic axis in GBM pathology

To comprehend how HIF1A biomarkers and their associated E3 ligases, as well as HAT enzymes and E2s, are involved, we have collated all of our research data. Table 4 demonstrates the strategy for choosing the dysregulated final axis in GBM. It revealed that Ube2E1 (K43), Ube2H (K8, K52) were connected with VHL enzymes and Ube2C (K18, K33), Ube2S (K168, K210, K211, K215, K216) linked with GNB2L1, while Ube2J2 (K64, K88) was associated with both VHL and GNB2L1 enzymes. Only a few of the predicted acetylation sites K8 of UBE2H, K33 of Ube2C, K198, K210, K211, K215, and K216 of Ube2S were verified with the MutPred2 predictor outcome “loss of acetylation site” following a single amino acid substitution mutation. The GBM was examined for each E2s connection with the HATs enzymes. Using the GEPIA2.0 program, the mRNA expression of each HATs enzyme was examined in a GBM patient sample. Out of all the enzymes, only HAT1 was connected to E2s at specific lysine residues. As a result, we suggested two novel pathways that may be therapeutic targets: HAT1-Ube2S(K211)-GNB2L1-HIF1A and HAT1-Ube2H(K8)-VHL-HIF1A. We anticipated a new route axis HAT1-Ube2S(K211)-GNB2L1-HIF1A implicated in the pathogenesis of GBM because K8 of Ube2H has already been identified in the literature (Lacoursiere et al., 2022). Thus, we predicted a new route axis, HAT1-Ube2S(K211)-GNB2L1-HIF1A, implicated in the etiology of GBM. We have demonstrated that in this pathway, HAT1 acetylates E2s, and Ube2S (a non-histone protein) at lysine residue K211 (near C-terminal), causing its overexpression. Numerous studies have demonstrated that non-histone protein acetylation is one of the critical factors influencing gene transcription. Alaei et al. (2018) found that the C-terminal acetylation of lysine modulates protein turnover and stability (Alaei et al., 2018). In contrast, early research showed that ubiquitin-mediated protein degradation could be stopped when the N-terminal-amino group is acetylated, and this degradation can happen to proteins with free-amino groups. Several signaling pathways along with the cell cycle can be regulated by protein acetylation (Hwang et al., 2010; Zhuang, 2013; You et al., 2022). Most HATs have a nucleus-specific location and operate as co-activators of transcription. The degradation of proteins is also connected to protein acetylation (Sterner and Berger, 2000; Varshavsky, 2019). Acetylation is a modification that can significantly modify a protein’s function by changing its hydrophobicity, solubility, and surface characteristics. These changes may impact the protein’s conformation and interactions with substrates, cofactors, and other macromolecules (Christensen et al., 2019b). As a result, C-terminal acetylation controls lysine’s ubiquitination and impacts its turnover. We postulated that acetylation of Ube2S at position 211, near the protein’s C-terminus, promotes and regulates GNB2L1’s protein turnover and ubiquitination modification. As a result of increased protein aggregation, the ability of GNB2L1 to ubiquitinate HIF1A is reduced, which further increases the expression level of the HIF1A protein (prevents its degradation by the UPS system).

**TABLE 4:** Correlation and expression analysis of HAT enzymes and prediction of therapeutic axis In GBM.

E3 ligase	E2 conjugating enzymes	Potential K residue position	Histone acetyltransferases (HATs) enzymes						Therapeutic axis	Loss of acetylation site
		Confidence score >2.5	CREBBP	EP300	HAT1	KAT2A	KAT2B	KAT5		
VHL	UBE2E1	43	-	-	√	χ	-	-	HAT1-UBE2E1(K43)-VHL	No
	UBE2H	8	-	-	√	-	-	-	HAT1-UBE2H(K8)-VHL	Yes
		52	-	-	√	-	-	-	HAT1-UBE2H(K52)-VHL	No
	UBE2J2	64	-	-	√	-	-	-	HAT1-UBE2J2(K64)-VHL	No
		88	-	-	√	-	-	χ	HAT1-UBE2J2(K88)-VHL	No
GNB2L1	UBE2C	18	-	χ	√	-	-	-	HAT1-UBE2C(K18)-GNB2L1	No
		33	-	-	-	-	χ	-	-	Yes
	UBE2J2	64	-	-	√	-	-	-	HAT1-UBE2J2(K64)-GNB2L1	No
		88	-	-	√	-	-	χ	HAT1-UBE2J2(K88)-GNB2L1	No
	UBE2S	198	-	-	-	χ	-	-	-	Yes
		210	-	-	-	χ	-	-	-	Yes
		211	-	-	√	χ	χ	-	HAT1-UBE2S(K211)-GNB2L1	Yes
		215	-	-	-	χ	-	-	-	Yes
		216	χ	-	-	χ	χ	-	-	Yes

⁃ Lysine residues marked in **blue are novel** and have not been previously documented in the literature for acetylation modification in GBM, patients.

⁃ *p*-value≤0.05: significant; *p*-value>0.05; ns: not significant.

⁃ √: signifies HAT1 enzymes expression is upregulated, with the significant positive correlation between HAT1 and Ube2E1, Ube2H and Ube2C, Ube2J2, Ube2S.

⁃ χ: signifies KAT2A enzyme expression is downregulated, with a not significant association between KAT2A and Ube2E1, Ube2A.

⁃ χ: signifies CREBBP, EP300, KAT2B, and KAT5 enzyme expression is not significant, with no significant association between CREBBP, and Ube2S; EP300 and Ube2C; KAT2B and Ube2C, Ube2S; KAT5 and Ube2J2.

⁃ The pink rectangle box represents the first proposed therapeutic axis in GBM.

⁃ The brown rectangle box represents the second proposed therapeutic axis in GBM.

Overexpressed Ube2S is linked with increased GNB2L1 and elevated HIF1A substrate. As per earlier research, acetylation is essential for p53 activation because it prevents the ubiquitin E3 ligase Mdm2 from inhibiting its ability to bind p53 for ubiquitination and proteasomal destruction. According to the theory of inter-protein acetylation-ubiquitination crosstalk, acetylation of Mdm2 by p300/CBP may prevent p53 from being subsequently ubiquitinated, increasing p53’s stability and transcriptional activity (Wang et al., 2004). Additionally, Sirt1’s ubiquitination and degradation may control the acetylation status of the histones in the downstream region, which would further epigenetically restrict the expression of the autophagy gene and encourage the spread of colorectal cancer (Shen et al., 2018).

**FIGURE 5:**
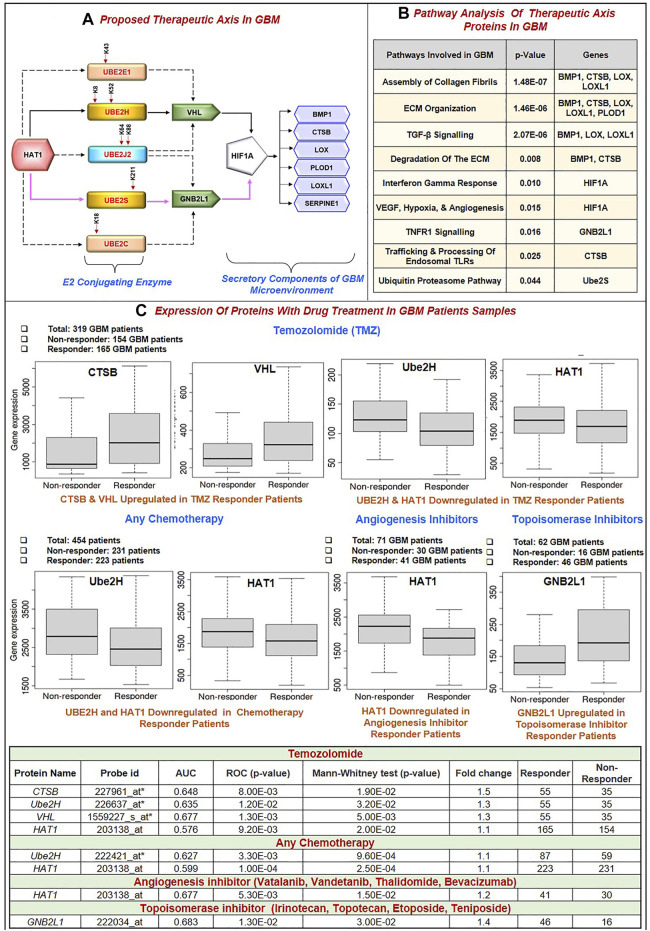
**(A)** Proposed Therapeutic axis: Based on our findings, two axes were proposed. First, there was HAT1-Ube2S(K211)-GNB2L1-HIF1A-BMP1/CTSB/LOX/LOXL1/PLOD1/SERPINE1. In this process, HAT1 will acetylate lysine residues at the 211* positions of Ube2S conjugating enzymes. This increases transcription and upregulation, linked to GNB2L1, an E3 ligase that regulates HIF1A activity in GBM. HIF1A overexpression links with the identified poor prognosis markers BMP1, CTSB, LOX, LOXL1, PLOD1, and SERPINE1. A solid pink colored line represents this axis. Second, HAT1-Ube2H(K8, K52)-VHL-HIF1A-BMP1/CTSB/LOX/LOXL1/PLOD1/SERPINE1 is involved. A solid black colored line represents this axis. HAT1 acetylates Lysine residues at K8 and K52* positions, and its overexpression has been linked to VHL, an E3 ligase, and HIF1A. This axis has been marked with a solid black line. Other therapeutic axes involving Ube2J2, Ube2E1 and VHL ligase, Ube2C and Ube2J2, and GNB2L1 ligase are possible, as illustrated in the figure with the dashed black line. * Signifies novel acetylation site on lysine residue. **(B)** Pathway analysis of the therapeutic axis’s protein showed genes involved in signaling pathways such as assembly of collagen fibrils, ECM organization, ECM degradation, Interferon-gamma response, hypoxia and angiogenesis, TNF signaling and ubiquitin-proteasome pathway. **(C)** Receiver operating characteristic (ROC) curve for biomarkers involved in therapeutic expression in Glioblastoma Multiforme. Area Under Curve (AUC) of time-dependent ROC curves verified the prognostic performance of the responder cohort after 16 months of treatment with Temozolomide (TMZ), chemotherapy, Angiogenesis, and Topoisomerase Inhibitors. The therapeutic axis includes HAT1, E2 enzymes (Ube2H, Ube2S, Ube2E1, Ube2C, Ube2J2), E3 ligase (VHL, GNB2L1), Prognosis markers (BMP1, CTSB, LOX, LOXL1, PLOD1 and SERPINE1). **(A)** In the TMZ responder cohort: CTSB and VHL expression was upregulated, and Ube2H and HAT1 were downregulated. **(B)** Chemotherapy responder cohort: HAT1 and Ube2H were downregulated. **(C)** Angiogenesis inhibitor responder cohort: HAT1 downregulated **(D)** Topoisomerase Inhibitors responder cohort: GNB2L1 upregulated in the responder. Tables show significant AUC and fold change expression between responder and non-responder patients to drug treatment.

Further, this significantly correlates with the GBM biomarkers BMP1, CTSB, LOX, LOXL1, PLOD1, and SERPINE1. Critical biological pathways, such as canonical and noncanonical TGF signaling, are regulated by BMP1, LOX, and LOXL1. Figure 5A illustrates the putative therapeutic axis and its influence on biological pathways in GBM. According to studies, TGF signaling regulates VEGF expression through SMAD-dependent signaling, which is crucial for angiogenesis in GBM. It contributes to the pathophysiology of tumors by controlling tumor growth, maintaining GSCs, and suppressing anti-tumor immunity (Lin et al., 2010; Sachdeva et al., 2019; Yu H. et al., 2020). Besides this, extracellular secreted CTSB can modify the TME through various non-cellular components and degrade the ECM. Cathepsins are a crucial class of proteins that are involved in the growth and propagation of cancer since they also interfere with the cell-cell adhesion molecules which encourage cell invasion and metastasis (Ding et al., 2022). Additionally, each contributes to the formation of collagen fibrils in the ECM. The normal brain contains minimal collagen, but it has been found that collagen gene expression is elevated in GBMs (Pointer et al., 2017). Moreover, LOX and LOXL1 isoforms are cleaved by BMP1-related proteases implies that these enzymes are matrix-oriented enzymes and possess strong binding with other ECM components including fibronectin, fibulin-4 and fibulin-5, and tropoelastin. In fact, research has revealed that inactivating the Lox and Loxl1 genes in mice models causes severe vascular problems because it disrupts the development of elastic fibers (Yang et al., 2020). Figure 5B depicts the study of different biological pathways of biomarkers associated with the proposed treatment axis in GBM. According to our findings, these expected axes in GBM may be targeted in GBM patient samples, which show that all proteins and enzymes associated with these pathways are noticeably enhanced at both the transcriptional and proteomic levels. Furthermore, they significantly connect with the appropriate partner proteins in GBM. So, we identified strategies that may be used to block the development of GBM.

### 3.9 Characterization of putative biomarkers involved in the proposed therapeutic axis in GBM

#### 3.9.1 Predictive markers response to GBM treatment

Despite advances in the molecular characterization of GBM, only a handful of predictive biomarkers exist with limited clinical relevance. We embraced the receiver operator characteristic (ROC) plotter webtool to link with protein expression amongst our proposed therapeutic axis in GBM tumor samples with therapies including temozolomide (TMZ), chemotherapy, Angiogenesis inhibitor (including Vatalanib, Vandetanib, Thalidomide, Bevacizumab) and topoisomerase inhibitors (including Irinotecan, Topotecan, Etoposide, Teniposide). For each protein, HAT1, Ube2E1, Ube2H, Ube2J2, Ube2S, Ube2C, VHL, GNB2L1, HIF1A, BMP1, CTSB, LOX, LOXL1, PLOD1, and SERPINE1, the expression was compared between responders and non-responder’s patients’ data with a Mann–Whitney U-test and area under curve (AUC). In response to TMZ, we discovered the enhanced expression of CTSB (AUC = 0.648) and VHL (AUC = 0.667). In response to TMZ and chemotherapy, it was shown that the expression of Ube2H (AUC = 0.635, 0.627 respectively) and HAT1 (AUC = 0.576, 0.599 respectively) had decreased.

Additionally, HAT1 expression was downregulated in angiogenesis inhibitor treatment responders (AUC = 0.677). In addition, patients who responded well to topoisomerase inhibitor medication had increased expression of GBN2L1 (AUC = 0.683). Hu et al. (2020) discovered YWHAB, PPAT, and NOL10 as novel biomarkers and validated their diagnostic and prognostic value for Hepatocellular carcinoma, and Zhang et al. (2020) found ELANE, GPX4, GSDMD, and TIRAP as a prognosis marker in Endometrial Cancer using ROC plotter tool (Hu et al., 2020; Zhang and Yang, 2021). Therefore, based on our findings, it can be concluded that CTSB, VHL, GNB2L1, Ube2H, and HAT1 have the potential to serve as candidates for predictive markers of response, provide a framework for preclinical investigations and perhaps improve patient classification for GBM in the future (Figure 5C).

#### 3.9.2 Correlation of therapeutic axis with top mutated genes in GBM

**FIGURE 6:**
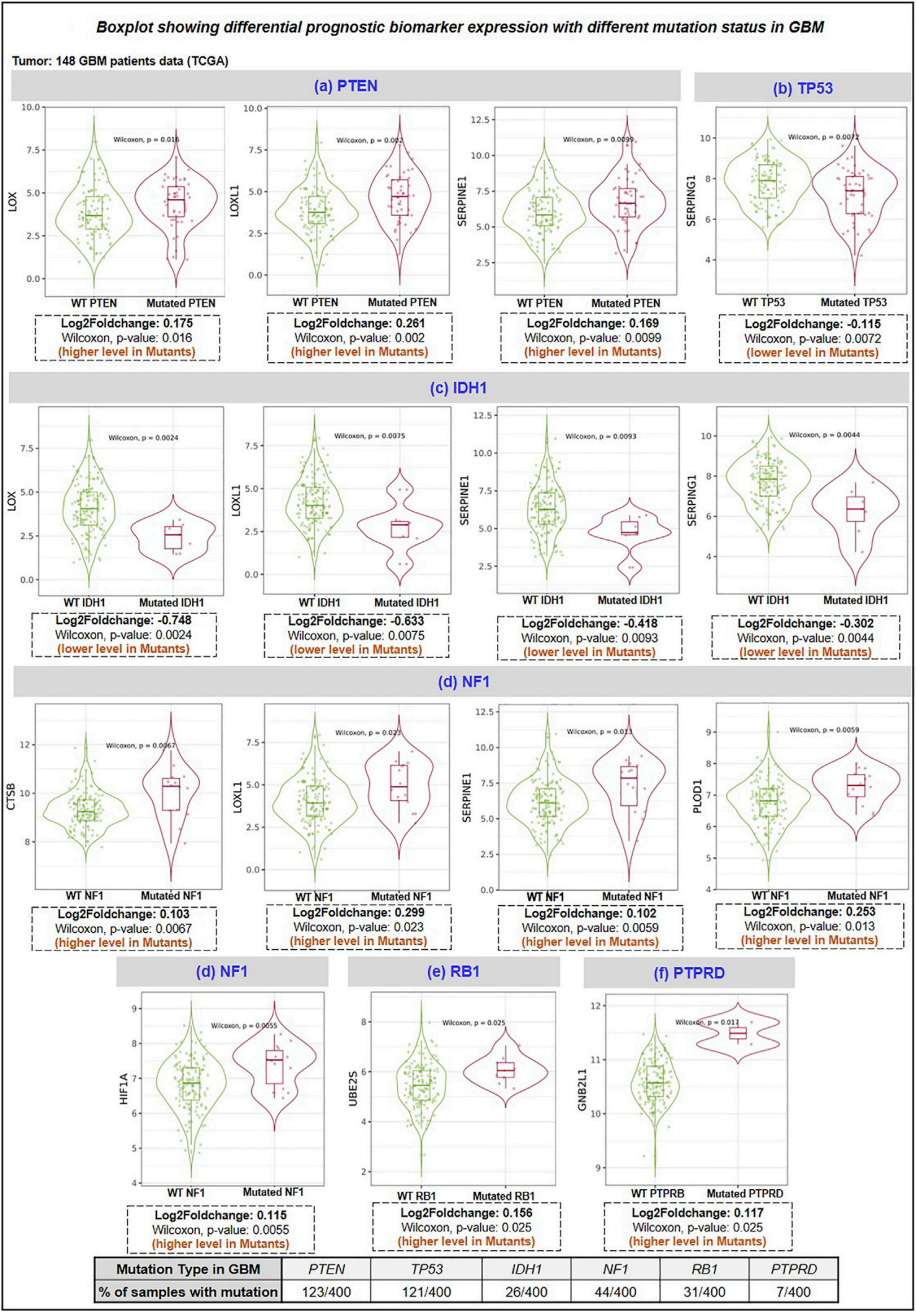
Differentiational expression analysis of prognosis biomarker with a top mutation in GBM. HAT1, E2 enzymes (Ube2H, Ube2S, Ube2E1, Ube2C, UbeJ2), E3 ligase (VHL, GNB2L1), Prognosis markers (BMP1, CTSB, LOX, LOXL1, PLOD1 and SERPING1) **(A)** PTEN mutation: LOX, LOXL1 and SERPINE1 were upregulated in GBM mutant group, **(B)** TP53 mutation: SERPING1 were downregulated in mutant GBM group, **(C)** IDH1 mutation: LOX, LOXL1, SERPINE1 and SERPING1 downregulated in the mutant group, **(D)** NF1 mutation: CTSB, LOXL1, SERPINE1, PLOD1 and HIF1A were upregulated in the mutant group. **(E)** RB1 mutation: Ube2S was upregulated, and **(F)** PTPRD: GNB2L1 was upregulated in the mutant group. PTPRD: Protein Tyrosine Phosphatase Receptor Type D; NF1: neurofibromin-1; RB1: Retinoblastoma gene; IDH1: isocitrate dehydrogenase 1 gene; PTEN: phosphatase and tensin homolog.

Here, we studied the differential expression of all proteins involved in the proposed therapeutic axis (HAT1, Ube2E1, Ube2H, Ube2J2, Ube2S, VHL, GNB2L1, HIF1A) along with prognostic biomarker (BMP1, CTSB, LOX, LOXL1, PLOD1, MMP9, SERPINE1, SERPING1) with top 10 genes mutated genes in GBM using “gene_module” tool of TIMER2.0 webserver. Research evidence suggests that the top 10 mutated genes in GBM are PTEN, TP53, EGFR, PIK3R1, PIK3CA, NF1, RB1, IDH1, PTPRD, and ERBB2 (Liu A. et al., 2016; Zhang et al., 2019). The incidence rate of each mutation in 400 GBM patient samples has been shown as PTEN (30.75%), TP53 (30.25%), EGFR (23.5%), NF1 (11%), PIK3CA (8.75%), PIK3R1 (8.5%), RB1 (7.75%), IDH1 (6.5%), PTPRD (1.75%), ERBB2 (1.25%). The expression of the interested protein was compared between GBM patients (n = 148) with wild-type and mutant-type genes. We have observed that GBM patient samples having a) PTEN mutation have higher expression of LOX, LOXL1, SERPINE1 protein, b) p53 mutation have decreased levels of SERPING1, c) IDH1 mutation have decreased levels of LOX, LOXL1, SERPINE1 and SERPING1, d) NF1 mutation have higher levels of CTSB, LOXL1, SERPINE1, PLOD1 and HIF1A, e) RB1 mutation have higher levels of Ube2S, f) PTPRD mutation have higher levels of GNB2L1. Figure 6 shows the boxplot of all significant biomarkers regulated with mutated genes in GBM.

#### 3.9.3 Association with human protein kinases in GBM

**FIGURE 7:**
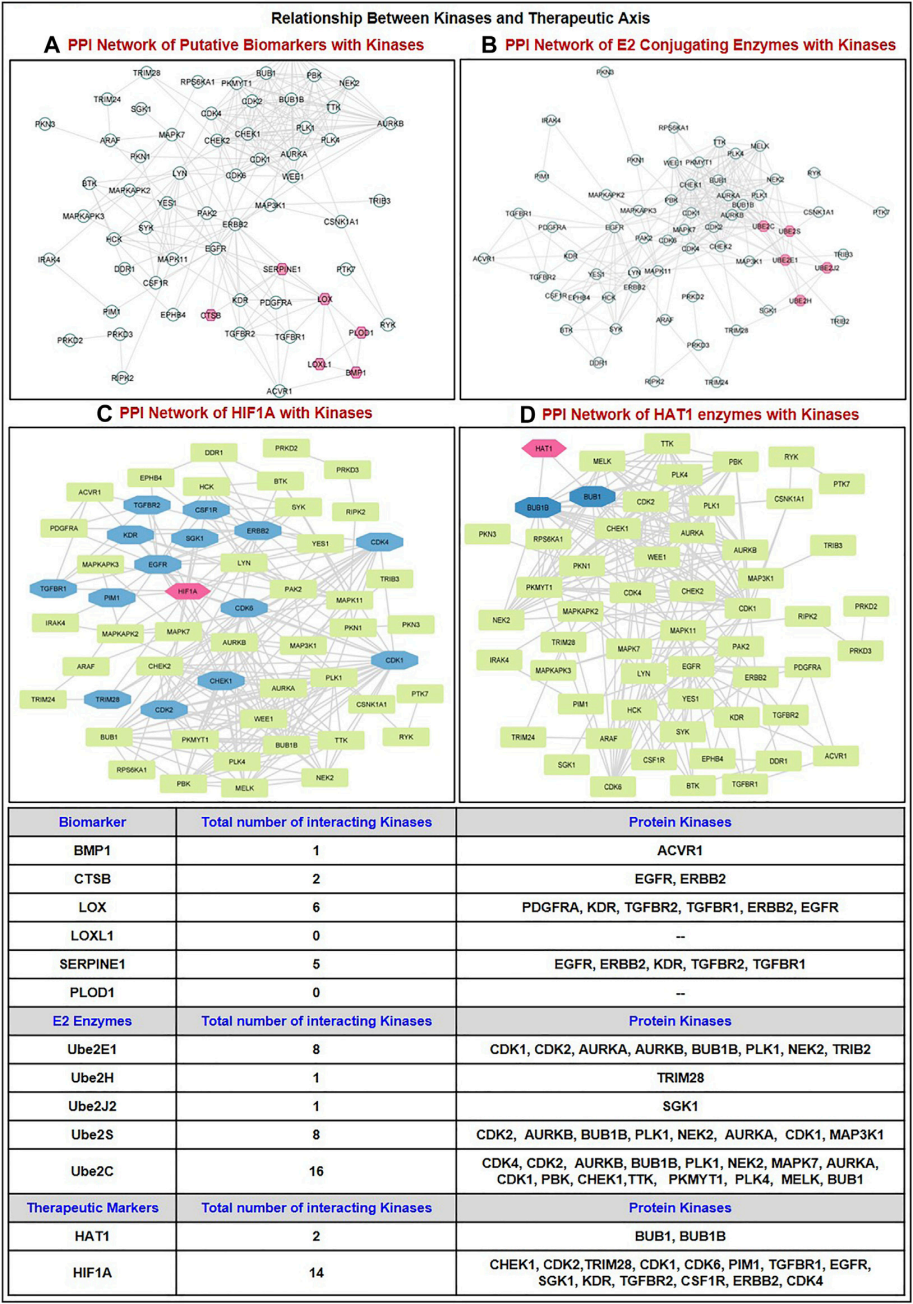
Correlation of dysregulated protein kinases (upregulated in GBM patient tumor samples) with the proteins involved in the proposed therapeutic axis. PPI network of kinases with **(A)** Putative biomarkers (BMP1, CTSB, LOX, LOXL1, PLOD1, SERPINE1); **(B)** E2s conjugating enzymes (Ube2S, Ube2H, and others Ube2E1, Ube2C, Ube2J2); **(C)** HIF1A; **(D)** HAT1 enzymes. GBM: Glioblastoma multiforme; PPI: Protein-Protein Interaction.

We have studied the expression of 536 human protein kinases in GBM and showed that 71 kinases were upregulated and 46 kinases were downregulated. Using protein-protein network analysis, we have studied the interaction between biomarkers (BMP1, CTSB, LOX, LOXL1, PLOD1, SERPINE1) with dysregulated kinases. We have shown (Figure 7A) LOX interacts with PDGFRA, KDR, TGFBR2, TGFBR1, ERBB2, EGFR; b) SERPINE1 interacts with EGFR, ERBB2, KDR, TGFBR2, TGFBR1; c) CTSB interact with EGFR, ERBB2, and d) BMP1: ACVR1. In addition, we have discussed the protein-protein interaction (PPI) between E2s with kinases and showed that the proposed E2s Ube2S interact with 8 kinases including CDK2, AURKB, BUB1B, PLK1, NEK2, AURKA, CDK1, MAP3K1 whereas Ube2H interact only with TRIM28 kinases (Figure 7B). Further the association of kinases with HIF1A biomarker and HAT1 enzymes. Results shows HIF1A interact with only BUB1 and BUB1B kinases whereas HAT1 enzymes interact with 14 kinases, namely, CHEK1, CDK1, CDK2, CDK4, CDK6, PIM1, TGFBR1, EGFR, SGK1, KDR, TGFBR2, CSF1R, ERBB2, and TRIM28 (Figures 7C,D **).** Here, we have briefly discussed the crucial role kinases play in the pathogenesis of GBM. For example, prior research confirmed that CDKs such as CDK2, 4, and 6 are stimulated in GBM which increases proliferation, radio, and chemoresistance; thus, inhibiting these will increase chemosensitivity to TMZ (Wang et al., 2016; Cao et al., 2020). Enhanced BUB1/BUB1B expression encourages growth and proliferation, whereas TRIM28 induces GBM cells to go into an autophagic phase and is associated with a bad prognosis for GBM patients (Peng et al., 2019; Long et al., 2021). Additionally, AURKA inhibits FOXM1 ubiquitination and increases the development of GBM (Zhang P. et al., 2022). While ERBB2, a member of the EGF receptor family, regulates glioma cell proliferation, immunological response, and activation of downstream signaling cascades (Mei et al., 2021). Other studies demonstrated that around 60% of initial GBMs have EGFR amplification, and 23% of classical tumors have a particular EGFR-III mutation, which makes them excellent candidates for therapeutic intervention. In contrast, a recent study investigated how EGFR functions as a tumor suppressor in EGFR-amplified GBM that is controlled by EGFR ligands (Xu et al., 2017; Guo et al., 2022).

## 4 Conclusion

Together, our investigations offer fresh insights into the expression of secretory components and their prognostic significance in the pathogenesis of the GBM microenvironment. In GBM patient samples, 8 elevated biomarkers, such as BMP1, CTSB, LOX, LOXL1, PLOD1, MMP9, SERPINE1, and SERPING1, were linked to poor prognosis in patients, and only BMP1, HIF1A, and TNFRSF1B, have been identified as substrates involved in the ubiquitination process corresponding E3 ligases. Only E3 ligase VHL and GNB2L1 recognize HIF1A was highly expressed after mRNA and protein levels were analyzed for expression. Interestingly, we found that the E2s Ube2C, Ube2E1, Ube2H, Ube2J2, Ube2L6, and Ube2S are highly expressed in GBM. After that, the correlation between E2s and VHL and GNB2L1 revealed a positive connection between VHL and Ube2E1, Ube2H, and Ube2J2 and GNB2L1 and Ube2C, Ube2J2, and Ube2S. Similarly, there was a significant association between VHL, and GNB2L1 with HIF1A. In addition, we have discovered all potential acetylation sites on the lysine residue of the E2s: UBE2C (12), Ube2E1 (15), Ube2H (13), Ube2J2 (15), and Ube2S (16). Only five E2s have confidence scores ≥2.5: K33 of Ube2C, K43 of Ube2E1, K8 and K52 of Ube2H, K64 and K88 of Ube2J2, and K198, K210, K211, K215, and K216.

According to the mutational analysis results, the acetylation site is lost due to a mutation at K33 of Ube2C or K8 of Ube2H with Q, L, R, or L. The Ube2S mutation causes the lack of acetylation at the corresponding “K" residue at K198 and K211 with L; at K210 and K216 with L, Q, and E; and K215 with L and Q. We have also discovered HATs enzymes that attack acetylated lysine residues in E2s. In GBM patient samples, we found that HAT1 positively correlated with the Ube2E1, Ube2H, Ube2J2, and Ube2S enzymes. In contrast, there is no correlation between HAT1 and Ube2C in GBM patient samples. Our study revealed that only HAT1 is overexpressed in GBM patient samples among the eight HAT enzymes. HAT1’s role as an oncogene is well known, and solid tumors, including esophageal, lung, liver, and pancreatic cancer, have been shown to overexpress the gene (Wu et al., 2019). After analyzing and collating all of the data from the study, we identified two pathways, one of which targeted either of the proteins’ components and the other, which was significantly active in GBM. HAT1-Ube2S(K211)-GNB2L1/HIF1A-BMP1/CTSB/LOX/LOXL1/PLOD1/SERPINE1 and HAT1-UbeH(K8)-VHL-HIF1A-BMP1/CTSB/LOX/LOXL1/PLOD1/SERPINE1 had high and medium confidence scores, respectively. HAT1 enzymes acetylate Ube2S’s 211-position lysine residue, increasing GNB2L1’s protein turnover while decreasing its ability to ubiquitinate its substrate HIF1A. This causes HIF1A to accumulate and overexpress itself in GBM. Being a transcription factor, HIF1A also controls the expression of BMP1, CTSB, LOX, LOXL1, PLOD1, and SERPINE1 indicators of poor prognosis in GBM. Major biological processes regulated by our identified axis were hypoxia, angiogenesis, ECM structure and degradation, EMT, IFN response, and TGF and TNF signaling. These signaling processes are essential to the pathophysiology of GBM. Therefore, we could target these cellular processes and reduce tumor burden by focusing on our identified therapeutic axis. We have also discovered the predictive markers CTSB and VHL for TMZ therapy, GNB2L1 for topoisomerase inhibitor therapy, Ube2H and HAT1 for TMZ and chemotherapy. HAT1 is also a hazard to angiogenesis inhibitors. The top 10 mutations already identified in GBM have been used to study alterations in the expression level of our therapeutic axis. Our work sheds light on the potential to investigate the use of secretory microenvironmental components in focusing on the GBM microenvironment. We have also demonstrated the protein-protein interaction between E2s with kinases and showed that the proposed E2s Ube2S interact with 8 kinases including CDK2, AURKB, BUB1B, PLK1, NEK2, AURKA, CDK1, MAP3K1 whereas Ube2H interact only with TRIM28 kinases. Thus, using computational and machine-learning-based tools and webservers to anticipate acetylation sites of E2s greatly facilitates the study of acetylation and saves valuable research time. More research and scientific studies are required to explore non-cellular components of the GBM microenvironment, PTM, especially acetylation, and E2s. However, the current study is accompanied by limitations, such as the small number of patient samples, *in vitro* and *in vivo* validation of biomarkers and acetylation sites, and lack of predictive biomarkers, substrates, and signaling molecules expression in GBM. Although, despite a computational study, the current study aims to bridge the gap between GBM, biomarkers, acetylation, and ubiquitination enzymes. The study opens the way for the researchers to validate the identified biomarkers in GBM therapeutics. Further, *in vitro* or *in vivo* validation of acetylating sites and ubiquitination factors (E3 ligases and E2 enzymes) through proteomic studies will lead to enhanced GBM therapeutics, which might cause an increased overall survival rate. Additionally, validation of identified therapeutic axis will have the potential to reverse the GBM etiology or help in drug discovery and development.

## Figures and Tables

**Table:** 

**GBM**	Glioblastoma Multiforme
**E2s**	E2 Conjugating Enzymes
**BBB**	Blood-Brain Barrier
**TME**	Tumor Microenvironment
**ECM**	Extracellular Matrix
**PTMs**	Post-Translational Modifications
**HATs**	Histone Acetyltransferases
**UPP**	Ubiquitin-Proteasome Pathway
**Ub**	Ubiquitin
**UPS**	Ubiquitin-Proteasome System
**GEPIA2.0**	Gene Expression Profiling Interactive Analysis
**GTEx**	Genotype-Tissue Expression
**TCGA**	The Cancer Genome Atlas
**RPPAs**	Reverse-Phase Protein Arrays
**CPTAC**	The National Cancer Institute’s Clinical Proteomic Tumor Analysis Consortium
**GO**	Gene Ontologies
**KEGG**	Kyoto Encyclopaedia Of Genes
**KM**	Kaplan-Meier
**OS**	Overall Survival
**DFS**	Disease-Free Survival
**GEO**	Gene Expression Omnibus
**HR**	Hazard Ratio
**CI**	Confidence Interval
**SVM**	Support Vector Machines
**UUCD**	Ubiquitin And Ubiquitin-Like Conjugation Database
**EVs**	Extracellular Vesicles
**BMP1**	Bone Morphogenetic Protein
**CTSB**	Cathepsin B
**LOX**	Lysyl Oxidase
**LOXL1**	Lysine Oxidase Like 1
**PLOD1**	Procollagen-Lysine,2-Oxoglutarate 5-Dioxygenase 1; Matrix Metallopeptidase 9
**SERPINE1**	Serpin Family E Member 1
**SERPING1**	Serine Protease Inhibitor Family G1
**VHL**	Von Hippel-Lindau
**FPR**	False Positive Rate
**ROC**	Receiver Operator Characteristic
**AUC**	Area Under Curve
**TMZ**	Temozolomide
**ROS**	Reactive oxygen species

## Data Availability

The original contributions presented in the study are included in the article/Supplementary Material, further inquiries can be directed to the corresponding author.
