# Supplementary material for: Identification and characterization of putative biomarkers and therapeutic axis in Glioblastoma multiforme microenvironment
Source: Front Cell Dev Biol. 2023 Jul 19;11:1236271. doi: 10.3389/fcell.2023.1236271 (PMC10395518; doi:10.3389/fcell.2023.1236271)
Supplement: Supplementary file 1 [file DataSheet1.docx]

**Identification and characterization of putative biomarkers and therapeutic axis in Glioblastoma multiforme microenvironment**

**Smita Kumari^1^ and Pravir Kumar^1,^**^†^

^1^Molecular Neuroscience and Functional Genomics Laboratory, Department of Biotechnology, Delhi Technological University (Formerly DCE), Delhi 110042

**^†^**Author to whom correspondence should be addressed**:**

**Pravir Kumar, M.Sc. (BHU), Ph.D. (Germany); PDF/Faculty (USA)**

Professor and Head, Department of Biotechnology

Dean, International Affairs, Delhi Technological University (Formerly Delhi College of Engineering)

Former Dean Alumni, Delhi Technological University (Formerly Delhi College of Engineering)

Former Faculty, Neurology Department, Tufts University School of Medicine, Boston, MA, USA

Editor*, Scientific Reports (Nature Publication)*

Molecular Neuroscience and Functional Genomics Laboratory

Shahbad Daulatpur, Bawana Road, Delhi 110042; Phone: +91- 9818898622

PubMed: https://www.ncbi.nlm.nih.gov/myncbi/1DW465XG9bp5p/bibliography/public/
[Google Scholar](https://scholar.google.co.in/citations?user=WVLI4i4AAAAJ&hl=en); [ORCID](https://orcid.org/0000-0001-7444-2344)

**S1**

**R2Q7 Supplementary Information Table S1:**

**Expression study of non-cellular secretory components of tumor microenvironment in Glioblastoma Multiforme**

| **Webtools** | | **RNA Sequence datasets** | | | | **Microarray datasets** | | | |
| --- | --- | --- | --- | --- | --- | --- | --- | --- | --- |
|  |  | ***GEPIA 2.0*** | ***UCSC XENA*** | | ***GLIOVIS*** | ***GLIOVIS*** | | ***TCGA_GBM*** | |
|  |  | **TCGA GBM_GTX** | **TCGA GBM** | **GDC TCGA GBA** | **TCGA RNA Sequence** | **REMBRANDT** | **GRAVENDEEL** | **HG-U133A** | **AGILENT-4502A** |
| **Chemokines** | XCL1 |  |  |  |  |  |  |  |  |
|  | CCL1 |  |  |  |  |  |  |  |  |
|  | CCL11 |  |  |  |  |  |  |  |  |
|  | CCL12 |  |  |  |  |  |  |  |  |
|  | CCL13 |  |  |  |  |  |  |  |  |
|  | CCL14 |  |  |  |  |  |  |  |  |
|  | CCL15 |  |  |  |  |  |  |  |  |
|  | CCL16 |  |  |  |  |  |  |  |  |
|  | CCL17 |  |  |  |  |  |  |  |  |
|  | CCL18 |  |  |  |  |  |  |  |  |
|  | CCL19 |  |  |  |  |  |  |  |  |
|  | CCL2 |  |  |  |  |  |  |  |  |
|  | CCL20 |  |  |  |  |  |  |  |  |
|  | CCL21 |  |  |  |  |  |  |  |  |
|  | CCL22 |  |  |  |  |  |  |  |  |
|  | CCL23 |  |  |  |  |  |  |  |  |
|  | CCL24 |  |  |  |  |  |  |  |  |
|  | CCL25 |  |  |  |  |  |  |  |  |
|  | CCL26 |  |  |  |  |  |  |  |  |
|  | CCL27 |  |  |  |  |  |  |  |  |
|  | CCL28 |  |  |  |  |  |  |  |  |
|  | CCL3 |  |  |  |  |  |  |  |  |
|  | CCL3L1 |  |  |  |  |  |  |  |  |
|  | CCL3L3 |  |  |  |  |  |  |  |  |
|  | CCL4 |  |  |  |  |  |  |  |  |
|  | CCL4L1 |  |  |  |  |  |  |  |  |
|  | CCL4L2 |  |  |  |  |  |  |  |  |

**S2**

|  | CCL5 |  |  |  |  |  |  |  |  |
| --- | --- | --- | --- | --- | --- | --- | --- | --- | --- |
|  | CCL6 |  |  |  |  |  |  |  |  |
|  | CCL7 |  |  |  |  |  |  |  |  |
|  | CCL8 |  |  |  |  |  |  |  |  |
|  | CCL9/10 |  |  |  |  |  |  |  |  |
|  | CX3CL1 |  |  |  |  |  |  |  |  |
|  | CXCL1 |  |  |  |  |  |  |  |  |
|  | CXCL10 |  |  |  |  |  |  |  |  |
|  | CXCL11 |  |  |  |  |  |  |  |  |
|  | CXCL12 |  |  |  |  |  |  |  |  |
|  | CXCL13 |  |  |  |  |  |  |  |  |
|  | CXCL14 |  |  |  |  |  |  |  |  |
|  | CXCL15 |  | | | | | | | |
|  | CXCL16 |  |  |  |  |  |  |  |  |
|  | CXCL17 |  |  |  |  |  |  |  |  |
|  | CXCL2 |  |  |  |  |  |  |  |  |
|  | CXCL22 |  |  |  |  |  |  |  |  |
|  | CXCL3 |  |  |  |  |  |  |  |  |
|  | CXCL4 |  |  |  |  |  |  |  |  |
|  | CXCL4L1 |  |  |  |  |  |  |  |  |
|  | CXCL5 |  |  |  |  |  |  |  |  |
|  | CXCL6 |  |  |  |  |  |  |  |  |
|  | CXCL7 |  |  |  |  |  |  |  |  |
|  | CXCL8 |  |  |  |  |  |  |  |  |
|  | CXCL9 |  |  |  |  |  |  |  |  |
|  | XCL2 |  |  |  |  |  |  |  |  |
|  | Activin |  |  |  |  |  |  |  |  |
|  | ADIPOQ |  |  |  |  |  |  |  |  |
|  | ANGPT1 |  |  |  |  |  |  |  |  |
|  | ANGPT2 |  |  |  |  |  |  |  |  |
|  | ANGPT4 |  |  |  |  |  |  |  |  |
|  | AREG |  |  |  |  |  |  |  |  |
|  | ARTN |  |  |  |  |  |  |  |  |
|  | Betacellulin |  |  |  |  |  |  |  |  |
|  | BFGF |  |  |  |  |  |  |  |  |
|  | BMP1 |  |  |  |  |  |  |  |  |
|  | BMP10 |  |  |  |  |  |  |  |  |
|  | BMP15 |  |  |  |  |  |  |  |  |
|  | BMP2 |  |  |  |  |  |  |  |  |
|  | BMP2a |  |  |  |  |  |  |  |  |
|  | BMP3 |  |  |  |  |  |  |  |  |
|  | BMP3b |  |  |  |  |  |  |  |  |
|  | BMP4 |  |  |  |  |  |  |  |  |
|  | BMP5 |  |  |  |  |  |  |  |  |
|  | BMP6 |  |  |  |  |  |  |  |  |
|  | BMP7 |  |  |  |  |  |  |  |  |
|  | BMP8 |  |  |  |  |  |  |  |  |
|  | BMP8a |  |  |  |  |  |  |  |  |
|  | BMP8b |  |  |  |  |  |  |  |  |
|  | BMP9 |  |  |  |  |  |  |  |  |
|  | BTC |  |  |  |  |  |  |  |  |
|  | CD38 |  |  |  |  |  |  |  |  |
|  | CD40LG |  |  |  |  |  |  |  |  |
|  | CD40LG |  |  |  |  |  |  |  |  |
|  | CD70 |  |  |  |  |  |  |  |  |

**S3**

|  | COL1a1 |  |  |  |  |  |  |  |  |
| --- | --- | --- | --- | --- | --- | --- | --- | --- | --- |
|  | COL1a2 |  |  |  |  |  |  |  |  |
|  | COL2a1 |  |  |  |  |  |  |  |  |
|  | Col3a1 |  |  |  |  |  |  |  |  |
|  | Col4a1 |  |  |  |  |  |  |  |  |
|  | Col4a2 |  |  |  |  |  |  |  |  |
|  | Col5a1 |  |  |  |  |  |  |  |  |
|  | Col5a2 |  |  |  |  |  |  |  |  |
|  | Col5a3 |  |  |  |  |  |  |  |  |
|  | Col7a1 |  |  |  |  |  |  |  |  |
|  | CSF1 |  |  |  |  |  |  |  |  |
|  | CSF2 |  |  |  |  |  |  |  |  |
|  | CSF3 |  |  |  |  |  |  |  |  |
|  | CTSB |  |  |  |  |  |  |  |  |
|  | DPP |  |  |  |  |  |  |  |  |
|  | EDA |  |  |  |  |  |  |  |  |
|  | EDA |  |  |  |  |  |  |  |  |
|  | EGF |  |  |  |  |  |  |  |  |
|  | Eln |  |  |  |  |  |  |  |  |
|  | EPGN |  |  |  |  |  |  |  |  |
|  | Epigen |  |  |  |  |  |  |  |  |
|  | EREG |  |  |  |  |  |  |  |  |
|  | Erythropoietin |  |  |  |  |  |  |  |  |
|  | FASLG |  |  |  |  |  |  |  |  |
|  | FGF1 |  |  |  |  |  |  |  |  |
|  | FGF10 |  |  |  |  |  |  |  |  |
|  | FGF16 |  |  |  |  |  |  |  |  |
|  | FGF17 |  |  |  |  |  |  |  |  |
|  | FGF18 |  |  |  |  |  |  |  |  |
|  | FGF19 |  |  |  |  |  |  |  |  |
|  | FGF2 |  |  |  |  |  |  |  |  |
|  | FGF20 |  |  |  |  |  |  |  |  |
|  | FGF21 |  |  |  |  |  |  |  |  |
|  | FGF22 |  |  |  |  |  |  |  |  |
|  | FGF23 |  |  |  |  |  |  |  |  |
|  | FGF3 |  |  |  |  |  |  |  |  |
|  | FGF4 |  |  |  |  |  |  |  |  |
|  | FGF5 |  |  |  |  |  |  |  |  |
|  | FGF6 |  |  |  |  |  |  |  |  |
|  | FGF7 |  |  |  |  |  |  |  |  |
|  | FGF8 |  |  |  |  |  |  |  |  |
|  | FGF9 |  |  |  |  |  |  |  |  |
|  | FLT3 |  |  |  |  |  |  |  |  |
|  | GDNF |  |  |  |  |  |  |  |  |
|  | HAS1 |  |  |  |  |  |  |  |  |
|  | HAS2 |  |  |  |  |  |  |  |  |
|  | HAS3 |  |  |  |  |  |  |  |  |
|  | HGF |  |  |  |  |  |  |  |  |
|  | HIF1A |  |  |  |  |  |  |  |  |
|  | HYAL1 |  |  |  |  |  |  |  |  |
|  | HYAL2 |  |  |  |  |  |  |  |  |
|  | HYAL3 |  |  |  |  |  |  |  |  |
|  | HYAL4 |  |  |  |  |  |  |  |  |
|  | IF01 |  |  |  |  |  |  |  |  |

**S4**

|  | IF010 |  |  |  |  |  |  |  |  |
| --- | --- | --- | --- | --- | --- | --- | --- | --- | --- |
|  | IF013 |  |  |  |  |  |  |  |  |
|  | IF014 |  |  |  |  |  |  |  |  |
|  | IF016 |  |  |  |  |  |  |  |  |
|  | IF017 |  |  |  |  |  |  |  |  |
|  | IF02 |  |  |  |  |  |  |  |  |
|  | IF04 |  |  |  |  |  |  |  |  |
|  | IF05 |  |  |  |  |  |  |  |  |
|  | IF06 |  |  |  |  |  |  |  |  |
|  | IF07 |  |  |  |  |  |  |  |  |
|  | IF08 |  |  |  |  |  |  |  |  |
|  | IFNb1 |  |  |  |  |  |  |  |  |
|  | IFNE |  |  |  |  |  |  |  |  |
|  | IFNg |  |  |  |  |  |  |  |  |
|  | IFNω/IFNW1 |  |  |  |  |  |  |  |  |
|  | IGF1 |  |  |  |  |  |  |  |  |
|  | IGF2 |  |  |  |  |  |  |  |  |
|  | IL10 |  |  |  |  |  |  |  |  |
|  | IL11 |  |  |  |  |  |  |  |  |
|  | IL12A |  |  |  |  |  |  |  |  |
|  | IL12B |  |  |  |  |  |  |  |  |
|  | IL13 |  |  |  |  |  |  |  |  |
|  | IL14 |  |  |  |  |  |  |  |  |
|  | IL15 |  |  |  |  |  |  |  |  |
|  | IL16 |  |  |  |  |  |  |  |  |
|  | IL17A |  |  |  |  |  |  |  |  |
|  | IL17B |  |  |  |  |  |  |  |  |
|  | IL17C |  |  |  |  |  |  |  |  |
|  | IL17D |  |  |  |  |  |  |  |  |
|  | IL17F |  |  |  |  |  |  |  |  |
|  | IL18 |  |  |  |  |  |  |  |  |
|  | IL18BP |  |  |  |  |  |  |  |  |
|  | IL19 |  |  |  |  |  |  |  |  |
|  | IL1a |  |  |  |  |  |  |  |  |
|  | IL1b |  |  |  |  |  |  |  |  |
|  | IL1F10 |  |  |  |  |  |  |  |  |
|  | IL1F5/IL36RN |  |  |  |  |  |  |  |  |
|  | IL1F6/IL36A |  |  |  |  |  |  |  |  |
|  | IL1F8/IL36B |  |  |  |  |  |  |  |  |
|  | IL1F9/IL36G |  |  |  |  |  |  |  |  |
|  | IL2 |  |  |  |  |  |  |  |  |
|  | IL20 |  |  |  |  |  |  |  |  |
|  | IL21 |  |  |  |  |  |  |  |  |
|  | IL22 |  |  |  |  |  |  |  |  |
|  | IL23 |  |  |  |  |  |  |  |  |
|  | IL24 |  |  |  |  |  |  |  |  |
|  | IL25 |  |  |  |  |  |  |  |  |
|  | IL26 |  |  |  |  |  |  |  |  |
|  | IL27 |  |  |  |  |  |  |  |  |
|  | IL28a/IFNL2 |  |  |  |  |  |  |  |  |
|  | IL28b/IFNL3 |  |  |  |  |  |  |  |  |
|  | IL29/IFNL1 |  |  |  |  |  |  |  |  |
|  | IL3 |  |  |  |  |  |  |  |  |
|  | IL30 |  |  |  |  |  |  |  |  |

**S5**

|  | IL31 |  |  |  |  |  |  |  |  |
| --- | --- | --- | --- | --- | --- | --- | --- | --- | --- |
|  | IL32 |  |  |  |  |  |  |  |  |
|  | IL33 |  |  |  |  |  |  |  |  |
|  | IL34 |  |  |  |  |  |  |  |  |
|  | IL35 |  |  |  |  |  |  |  |  |
|  | IL36a |  |  |  |  |  |  |  |  |
|  | IL36b |  |  |  |  |  |  |  |  |
|  | IL36g |  |  |  |  |  |  |  |  |
|  | IL37 |  |  |  |  |  |  |  |  |
|  | IL38/IL1F10 |  |  |  |  |  |  |  |  |
|  | IL4 |  |  |  |  |  |  |  |  |
|  | IL5 |  |  |  |  |  |  |  |  |
|  | IL6 |  |  |  |  |  |  |  |  |
|  | IL7 |  |  |  |  |  |  |  |  |
|  | IL8 |  |  |  |  |  |  |  |  |
|  | IL9 |  |  |  |  |  |  |  |  |
|  | KMO |  |  |  |  |  |  |  |  |
|  | Lama1 |  |  |  |  |  |  |  |  |
|  | Lama2 |  |  |  |  |  |  |  |  |
|  | Lama3 |  |  |  |  |  |  |  |  |
|  | Lama4 |  |  |  |  |  |  |  |  |
|  | Lama5 |  |  |  |  |  |  |  |  |
|  | Lamb1 |  |  |  |  |  |  |  |  |
|  | LeP |  |  |  |  |  |  |  |  |
|  | LEP (Leptin) |  |  |  |  |  |  |  |  |
|  | LGALS1 |  |  |  |  |  |  |  |  |
|  | LGALS12 |  |  |  |  |  |  |  |  |
|  | LGALS13 |  |  |  |  |  |  |  |  |
|  | LGALS14 |  |  |  |  |  |  |  |  |
|  | LGALS16 |  |  |  |  |  |  |  |  |
|  | LGALS2 |  |  |  |  |  |  |  |  |
|  | LGALS3 |  |  |  |  |  |  |  |  |
|  | LGALS4 |  |  |  |  |  |  |  |  |
|  | LGALS7 |  |  |  |  |  |  |  |  |
|  | LGALS8 |  |  |  |  |  |  |  |  |
|  | LGALS9 |  |  |  |  |  |  |  |  |
|  | LIF |  |  |  |  |  |  |  |  |
|  | LOX |  |  |  |  |  |  |  |  |
|  | LOXL1 |  |  |  |  |  |  |  |  |
|  | LOXL2 |  |  |  |  |  |  |  |  |
|  | LOXL3 |  |  |  |  |  |  |  |  |
|  | LOXL4 |  |  |  |  |  |  |  |  |
|  | LTA |  |  |  |  |  |  |  |  |
|  | LTB |  |  |  |  |  |  |  |  |
|  | MCSF |  |  |  |  |  |  |  |  |
|  | MIF |  |  |  |  |  |  |  |  |
|  | MMP1 |  |  |  |  |  |  |  |  |
|  | MMP10 |  |  |  |  |  |  |  |  |
|  | MMP11 |  |  |  |  |  |  |  |  |
|  | MMP12 |  |  |  |  |  |  |  |  |
|  | MMP13 |  |  |  |  |  |  |  |  |
|  | MMP14 |  |  |  |  |  |  |  |  |
|  | MMP15 |  |  |  |  |  |  |  |  |
|  | MMP16 |  |  |  |  |  |  |  |  |

**S6**

|  | MMP17 |  |  |  |  |  |  |  |  |
| --- | --- | --- | --- | --- | --- | --- | --- | --- | --- |
|  | MMP19 |  |  |  |  |  |  |  |  |
|  | MMP2 |  |  |  |  |  |  |  |  |
|  | MMP3 |  |  |  |  |  |  |  |  |
|  | MMP7 |  |  |  |  |  |  |  |  |
|  | MMP8 |  |  |  |  |  |  |  |  |
|  | MMP9 |  |  |  |  |  |  |  |  |
|  | MST1 |  |  |  |  |  |  |  |  |
|  | NRG1 |  |  |  |  |  |  |  |  |
|  | NRG2 |  |  |  |  |  |  |  |  |
|  | NRG3 |  |  |  |  |  |  |  |  |
|  | NRG4 |  |  |  |  |  |  |  |  |
|  | NRTN |  |  |  |  |  |  |  |  |
|  | Oncostatin M |  |  |  |  |  |  |  |  |
|  | PDGFA |  |  |  |  |  |  |  |  |
|  | PDGFB |  |  |  |  |  |  |  |  |
|  | PDGFC |  |  |  |  |  |  |  |  |
|  | PDGFD |  |  |  |  |  |  |  |  |
|  | PGE2 |  |  |  |  |  |  |  |  |
|  | PlGF |  |  |  |  |  |  |  |  |
|  | PLOD1 |  |  |  |  |  |  |  |  |
|  | PLOD2 |  |  |  |  |  |  |  |  |
|  | PLOD3 |  |  |  |  |  |  |  |  |
|  | Proepiregulin |  |  |  |  |  |  |  |  |
|  | Prolactin |  |  |  |  |  |  |  |  |
|  | PSPN |  |  |  |  |  |  |  |  |
|  | PTGES2 |  |  |  |  |  |  |  |  |
|  | ROS1 |  |  |  |  |  |  |  |  |
|  | SDF1 |  |  |  |  |  |  |  |  |
|  | SDF2 |  |  |  |  |  |  |  |  |
|  | SDF4 |  |  |  |  |  |  |  |  |
|  | SERPINE1 |  |  |  |  |  |  |  |  |
|  | SERPING1 |  |  |  |  |  |  |  |  |
|  | SPP1 |  |  |  |  |  |  |  |  |
|  | TGFb3 |  |  |  |  |  |  |  |  |
|  | TGFβ1 |  |  |  |  |  |  |  |  |
|  | TGFβ2 |  |  |  |  |  |  |  |  |
|  | TIMP1 |  |  |  |  |  |  |  |  |
|  | TIMP2 |  |  |  |  |  |  |  |  |
|  | TIMP3 |  |  |  |  |  |  |  |  |
|  | TIMP4 |  |  |  |  |  |  |  |  |
|  | TNF |  |  |  |  |  |  |  |  |
|  | TNFAIP2 |  |  |  |  |  |  |  |  |
|  | TNFAIP6 |  |  |  |  |  |  |  |  |
|  | TNFRSF1B |  |  |  |  |  |  |  |  |
|  | TNFSF10 |  |  |  |  |  |  |  |  |
|  | TNFSF11 |  |  |  |  |  |  |  |  |
|  | TNFSF12 |  |  |  |  |  |  |  |  |
|  | TNFSF13 |  |  |  |  |  |  |  |  |
|  | TNFSF13B |  |  |  |  |  |  |  |  |
|  | TNFSF14 |  |  |  |  |  |  |  |  |
|  | TNFSF15 |  |  |  |  |  |  |  |  |
|  | TNFSF15 |  |  |  |  |  |  |  |  |
|  | TNFSF18 |  |  |  |  |  |  |  |  |

**S7**

|  | TNFSF2 |  |  |  |  |  |  |  |  |
| --- | --- | --- | --- | --- | --- | --- | --- | --- | --- |
|  | TNFSF4 |  |  |  |  |  |  |  |  |
|  | TNFSF8 |  |  |  |  |  |  |  |  |
|  | TNFSF9 |  |  |  |  |  |  |  |  |
|  | VEGFA |  |  |  |  |  |  |  |  |
|  | VEGFB |  |  |  |  |  |  |  |  |
|  | VEGFC |  |  |  |  |  |  |  |  |
|  | VEGFD |  |  |  |  |  |  |  |  |
| **Patient samples number used in respective study** | | | | | | | | | |
| TUMOR | | 163 | 154 | 155 | 156 | 225 | 117 | 528 | 489 |
| N0N-TUMOR | | 207 | 5 | 5 | 4 | 28 | 8 | 10 | 10 |
| **Upregulated in GBM** | | | |  | **p<0.001** |  | **p<0.01** |  | **p<0.05** |
| **Downregulated in GBM** | | | |  | **p<0.001** |  | **p<0.01** |  | **p<0.05** |
| **Not significant** | | | | **p>0.05** | | | | | |

**Supplementary Information Table S2: Description Of 44 Biomarkers Dysregulated in Glioblastoma Multiforme**

| **Description of Genes** | |
| --- | --- |
| **Chemokines** | |
| CCL5 | C-C motif chemokine 5 |
| CX3CL1 | Fractalkine |
| CXCL16 | C-X-C motif chemokine 16 |
| **Cytokines and Growth factors** | |
| ANGPT2 | Angiopoietin-2 |
| BMP1 | Bone morphogenetic protein 1 |
| BMP7 | Bone morphogenetic protein 7 |
| COL1A1 | Collagen alpha-1(I) chain |
| COL1A2 | Collagen alpha-2(I) chain |
| COL3A1 | Collagen alpha-1(III) chain |
| COL4A1 | Collagen alpha-1(IV) chain |
| COL4A2 | Collagen alpha-2(IV) chain |
| COL5A1 | Collagen alpha-1(V) chain |
| COL5A2 | Collagen alpha-2(V) chain |
| CTSB | Cathepsin B |
| HIF1A | Hypoxia-inducible factor 1-alpha |
| IL-18 | Interleukin-18 |
| LAMA4 | Laminin subunit alpha-4 |
| LAMA5 | Laminin subunit alpha-5 |
| LAMB1 | Laminin subunit Beta-6 |
| LGALS3 | Galectin-3 |
| LGALS9 | Galectin-9 |
| LOX | Protein-lysine 6-oxidase |
| LOXL1 | Lysyl oxidase homolog 1 |
| LOXL3 | Lysyl oxidase homolog 3 |
| MMP14 | Matrix metalloproteinase-17 |
| MMP17 | Matrix metalloproteinase-15 |
| MMP2 | Matrix metalloproteinase-2 |
| MMP9 | Matrix metalloproteinase-9 |
| PLOD1 | Procollagen-lysine,2-oxoglutarate 5-dioxygenase 1 |
| PLOD2 | Procollagen-lysine,2-oxoglutarate 5-dioxygenase 2 |
| PLOD3 | Multifunctional procollagen lysine hydroxylase and glycosyltransferase LH3 |

**S8**

| PTGES2 | Prostaglandin E synthase 2 |
| --- | --- |
| SDF2 | Stromal cell-derived factor 2 |
| SDF4 | 45 kDa calcium-binding protein |
| SERPINE1 | Plasminogen activator inhibitor 1 |
| SERPING1 | Plasma protease C1 inhibitor |
| SPP1 | Osteopontin |
| TGFβ1 | Transforming growth factor beta-1 proprotein |
| TGFβ2 | Transforming growth factor beta-2 proprotein |
| TIMP1 | Metalloproteinase inhibitor 1 |
| TIMP3 | Metalloproteinase inhibitor 3 |
| TNFAIP6 | Tumor necrosis factor-inducible gene 6 protein |
| TNFRSF1B | Tumor necrosis factor receptor superfamily member 1B |
| VEGFA | Vascular endothelial growth factor A |

**S9**

**Supplementary Information Figure S1**


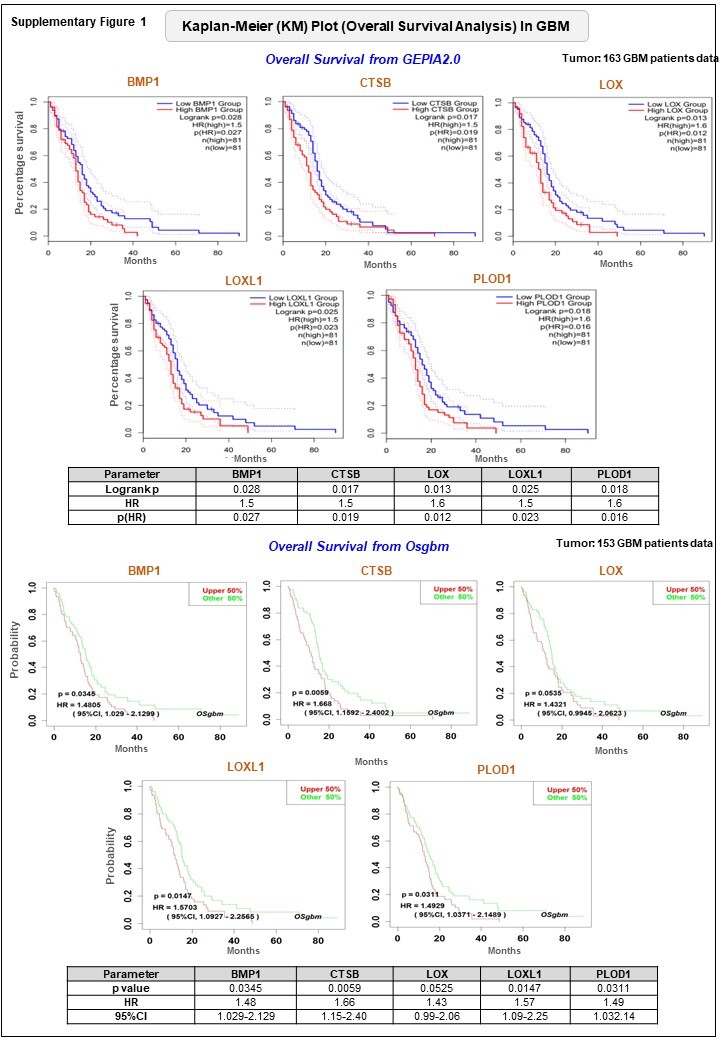


**Supplementary Information Figure S1: Kaplan-Meier (KM) plot for overall survival (OS) in GBM patient samples from TCGA datasets: OS time plotted through GEPIA2.0 and OSgbm between higher-expression-level and lower-expression-level tumors in GBM TCGA tumor types with shorter overall survival time and worse OS prognosis. Red line shows the cases with highly expressed biomarker and blue/green line is indicated for the cases with lowly expressed biomarker. HR: hazard ratio; p-value≤0.05**

**S10**

**Supplementary Information Figure S2**

**
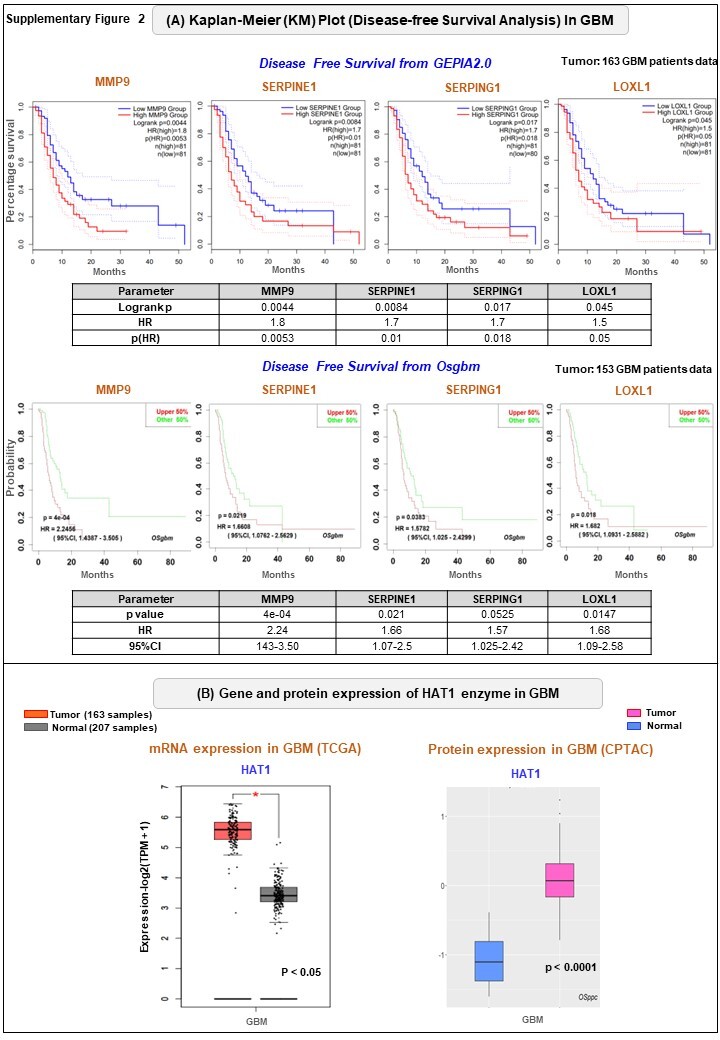
**

**Supplementary Information Figure S2: (A) Kaplan-Meier (KM) plot for Disease free survival (DFS) in GBM patient samples from TCGA datasets. (A) DFS time plotted through GEPIA2.0 and OSgbm between higher-expression-level and lower-expression-level tumor in the TCGA tumor types with worse prognosis. Red line shows the cases with highly expressed biomarker and blue/green line is indicated for the cases with lowly expressed biomarker. HR: hazard ratio, p-value≤0.05. (B) Gene and protein expression of HAT1 enzymes in GBM: Box plot reveals that the significantly over-expression of HAT1 enzymes in GBM tumor samples as compared to normal sample both at mRNA and protein level. Expression data was collected from GEPIA2.0 and Osppc tool.**

**S11**

**Supplementary Information Table S3: Physical Significance of** **E2 Conjugating Enzymes' Lysine (K) Residue Mutation Owing to A Single Amino Acid Substitution on Acetylation**

| **Ube2C_HUMAN** | | | | | | | |
| --- | --- | --- | --- | --- | --- | --- | --- |
| **Lysin Residue** | **Mutation substitution** | **Nature to mutation** | **Molecular mechanisms with p-values <= 0.05** | **MutPred2 Score** | **Probability** | **p-value** | **Affected PROSITE and ELM Motifs** |
| **K18** | Lys(K)-Leu(L) | Non-polar | Loss of Methylation at K18 | 0.772 | 0.49 | 1.10E-04 | ELME000102 |
|  |  |  | Loss of Ubiquitylation at K18 |  | 0.34 | 4.40E-05 |  |
|  |  |  | Loss of SUMOylation at K18 |  | 0.33 | 2.20E-03 |  |
|  |  |  | Altered Disordered interface |  | 0.28 | 4.00E-02 |  |
|  |  |  | Loss of ADP-ribosylation at R17 |  | 0.23 | 2.00E-02 |  |
|  |  |  | Loss of O-linked glycosylation at S23 |  | 0.13 | 4.00E-02 |  |
|  | Lys(K)-Gln(Q) | Polar | Loss of Methylation at K18 | 0.536 | 0.49 | 1.10E-04 | ELME000102 |
|  |  |  | Loss of Ubiquitylation at K18 |  | 0.34 | 4.40E-05 |  |
|  |  |  | Loss of SUMOylation at K18 |  | 0.33 | 2.20E-03 |  |
|  |  |  | Loss of ADP-ribosylation at R17 |  | 0.23 | 2.00E-02 |  |
|  |  |  | Loss of O-linked glycosylation at S23 |  | 0.13 | 4.00E-02 |  |
|  |  |  | Gain of Pyrrolidone carboxylic acid at K18 |  | 0.07 | 2.00E-02 |  |
|  | Lys(K)-Glu(E) | Negatively | Loss of Methylation at K18 | 0.612 | 0.49 | 1.10E-04 | ELME000102 |
|  |  |  | Loss of Ubiquitylation at K18 |  | 0.34 | 4.40E-05 |  |
|  |  |  | Loss of SUMOylation at K18 |  | 0.33 | 2.20E-03 |  |
|  |  |  | Loss of ADP-ribosylation at R17 |  | 0.24 | 2.00E-02 |  |
|  |  |  | Loss of O-linked glycosylation at S23 |  | 0.13 | 4.00E-02 |  |
|  |  |  | Gain of Helix |  | 0.28 | 2.00E-02 |  |
| **K33** | Lys(K)-Leu(L) | Non-polar | Loss of Intrinsic disorder | 0.908 | 0.41 | 2.00E-02 | ELME000093, ELME000100, ELME000108, PS00009 |
|  |  |  | Loss of Acetylation at K33 |  | 0.23 | 2.00E-02 |  |
|  |  |  | Loss of ADP-ribosylation at R28 |  | 0.21 | 3.00E-02 |  |
|  |  |  | Loss of Methylation at K33 |  | 0.1 | 4.00E-02 |  |
|  | Lys(K)-Gln(Q) | Polar | Loss of Acetylation at K33 | 0.804 | 0.23 | 2.00E-02 | ELME000093, ELME000100, ELME000108, ELME000193, PS00009 |
|  |  |  | Loss of ADP-ribosylation at R28 |  | 0.21 | 3.00E-02 |  |
|  |  |  | Loss of Methylation at K33 |  | 0.1 | 4.00E-02 |  |
|  | Lys(K)-Arg(R) | Positively | Loss of Acetylation at K33 | 0.681 | 0.23 | 2.00E-02 | ELME000012, ELME000093, ELME000100, ELME000102, ELME000108, PS00009 |
|  |  |  | Loss of ADP-ribosylation at R28 |  | 0.21 | 3.00E-02 |  |
|  |  |  | Loss of Methylation at K33 |  | 0.1 | 5.00E-02 |  |
|  | Lys(K)-Glu(E) | Negatively | Loss of Acetylation at K33 | 0.868 | 0.23 | 2.00E-02 | ELME000093, ELME000100, ELME000108, ELME000193, PS00009 |
|  |  |  | Gain of ADP-ribosylation at R28 |  | 0.22 | 2.00E-02 |  |
|  |  |  | Loss of Methylation at K33 |  | 0.1 | 4.40E-05 |  |
|  |  |  |  |  |  |  |  |
| **Ube2E1_HUMAN** | | | | | | | |
| **K43** | Lys(K)-Leu(L) | Non-polar | Altered Disordered interface | 0.562 | 0.38 | 7.90E-03 | ELME000053, ELME000173, ELME000333, ELME000335, ELME000336 |
|  |  |  | Loss of Ubiquitylation at K43 |  | 0.17 | 2.00E-02 |  |
|  |  |  | Loss of Methylation at K40 |  | 0.09 | 5.00E-02 |  |

**S12**

| **Ube2H_HUMAN** | | | | | | | |
| --- | --- | --- | --- | --- | --- | --- | --- |
| **K8** | Lys(K)-Leu(L) | Non-polar | Loss of Intrinsic disorder | 0.827 | 0.47 | 1.00E-02 | ELME000012, ELME000063, ELME000093, ELME000100, ELME000108, ELME000153, ELME000159, PS00009 |
|  |  |  | Altered Ordered interface |  | 0.28 | 4.00E-02 |  |
|  |  |  | Loss of B-factor |  | 0.28 | 2.00E-02 |  |
|  |  |  | Loss of Acetylation at K8 |  | 0.24 | 2.00E-02 |  |
|  |  |  | Altered DNA binding |  | 0.21 | 1.00E-02 |  |
|  |  |  | Loss of Ubiquitylation at K8 |  | 0.18 | 2.00E-02 |  |
|  |  |  | Loss of Methylation at K8 |  | 0.12 | 3.00E-02 |  |
|  | Lys(K)-Gln(Q) | Polar | Loss of B-factor | 0.758 | 0.26 | 4.00E-02 | ELME000063, ELME000093, ELME000100, ELME000108, ELME000153, ELME000159, PS00009 |
|  |  |  | Loss of Acetylation at K8 |  | 0.24 | 2.00E-02 |  |
|  |  |  | Altered DNA binding |  | 0.2 | 2.00E-02 |  |
|  |  |  | Loss of Ubiquitylation at K8 |  | 0.18 | 2.00E-02 |  |
|  |  |  | Loss of Methylation at K8 |  | 0.12 | 3.00E-02 |  |
|  | Lys(K)-Arg(R) | Positively | Gain of Helix | 0.661 | 0.27 | 5.00E-02 | ELME000012, ELME000061, ELME000063, ELME000093, ELME000100, ELME000108, ELME000153, ELME000159, PS00009 |
|  |  |  | Loss of Acetylation at K8 |  | 0.24 | 2.00E-02 |  |
|  |  |  | Altered DNA binding |  | 0.21 | 2.00E-02 |  |
|  |  |  | Loss of Ubiquitylation at K8 |  | 0.18 | 2.00E-02 |  |
|  |  |  | Loss of Methylation at K8 |  | 0.12 | 3.00E-02 |  |
|  | Lys(K)-Glu(E) | Negatively | Loss of Acetylation at K8 | 0.831 | 0.24 | 2.00E-02 | ELME000063, ELME000064, ELME000093, ELME000100, ELME000108, ELME000153, ELME000159, PS00006, PS00009 |
|  |  |  | Altered DNA binding |  | 0.2 | 2.00E-02 |  |
|  |  |  | Loss of Ubiquitylation at K8 |  | 0.18 | 2.00E-02 |  |
|  |  |  | Loss of Methylation at K8 |  | 0.12 | 3.00E-02 |  |
| **K52** | Lys(K)-Leu(L) | Non-polar | Loss of Relative solvent accessibility | 0.943 | 0.4 | 7.50E-04 | ELME000047, ELME000155, ELME000333 |
|  |  |  | Altered Ordered interface |  | 0.34 | 7.60E-03 |  |
|  |  |  | Altered Transmembrane protein |  | 0.29 | 1.90E-04 |  |
|  |  |  | Altered Metal binding |  | 0.28 | 6.40E-03 |  |
|  |  |  | Loss of Allosteric site at W51 |  | 0.26 | 1.00E-02 |  |
|  | Lys(K)-Gln(Q) | Polar | Loss of Relative solvent accessibility | 0.894 | 0.39 | 1.00E-03 | ELME000155 |
|  |  |  | Altered Metal binding |  | 0.27 | 7.80E-03 |  |
|  |  |  | Loss of Allosteric site at W51 |  | 0.27 | 8.90E-03 |  |
|  |  |  | Altered Transmembrane protein |  | 0.25 | 1.60E-03 |  |
|  |  |  | Altered Ordered interface |  | 0.25 | 2.00E-02 |  |
|  | Lys(K)-Arg(R) | Positively | Loss of Relative solvent accessibility | 0.821 | 0.33 | 4.10E-03 | ELME000012, ELME000155 |
|  |  |  | Altered Ordered interface |  | 0.29 | 3.00E-02 |  |
|  |  |  | Altered Metal binding |  | 0.27 | 8.80E-03 |  |
|  |  |  | Altered Transmembrane protein |  | 0.27 | 6.50E-04 |  |
|  |  |  | Gain of Allosteric site at W51 |  | 0.26 | 7.80E-03 |  |
|  | Lys(K)-Glu(E) | Negatively | Altered Ordered interface | 0.924 | 0.29 | 3.00E-02 | ELME000155 |
|  |  |  | Altered Transmembrane protein |  | 0.28 | 6.60E-04 |  |
|  |  |  | Loss of Relative solvent accessibility |  | 0.28 | 2.00E-02 |  |
|  |  |  | Loss of Allosteric site at W51 |  | 0.27 | 9.40E-03 |  |
|  |  |  | Altered Metal binding |  | 0.26 | 1.00E-02 |  |

**S13**

| **Ube2J2_HUMAN** | | | | | | | |
| --- | --- | --- | --- | --- | --- | --- | --- |
| **K64** | Lys(K)-Leu(L) | Non-polar | Altered Transmembrane protein | 0.704 | 0.3 | 1.50E-04 | ELME000120, ELME000137, ELME000146, ELME000317 |
|  |  |  | Altered Metal binding |  | 0.26 | 6.70E-03 |  |
|  |  |  | Altered Ordered interface |  | 0.25 | 0.02 |  |
|  |  |  | Gain of Sulfation at Y60 |  | 0.02 | 0.02 |  |
|  | Lys(K)-Gln(Q) | Polar | Altered Transmembrane protein | 0.504 | 0.3 | 1.60E-04 | ELME000137, ELME000146, ELME000163, ELME000317 |
|  |  |  | Altered Metal binding |  | 0.25 | 8.20E-03 |  |
|  |  |  | Altered Ordered interface |  | 0.25 | 0.02 |  |
|  |  |  | Gain of Sulfation at Y60 |  | 0.02 | 0.03 |  |
|  | Lys(K)-Glu(E) | Negatively | Altered Transmembrane protein | 0.59 | 0.31 | 1.20E-04 | ELME000137, ELME000146, ELME000317 |
|  |  |  | Altered Metal binding |  | 0.24 | 9.70E-03 |  |
|  |  |  | Altered Ordered interface |  | 0.24 | 4.00E-02 |  |
|  |  |  | Gain of Sulfation at Y60 |  | 0.03 | 2.00E-02 |  |
| **K88** | Lys(K)-Leu(L) | Non-polar | Loss of Strand | 0.538 | 0.26 | 4.00E-02 | ELME000233, ELME000336 |
|  | Lys(K)-Gln(Q) | Polar | Gain of Strand | 0.704 | 0.26 | 4.00E-02 | ELME000233 |
|  | Lys(K)-Glu(E) | Negatively | Gain of Loop | 0.812 | 0.29 | 1.00E-02 | ELME000233 |
|  |  |  | Gain of Strand |  | 0.26 | 4.00E-02 |  |
| **Ube2S_HUMAN** | | | | | | | |
| **K198** | Lys(K)-Leu(L) | Non-polar | Loss of Acetylation at K198 | 0.567 | 0.43 | 9.80E-04 | None |
|  |  |  | Loss of SUMOylation at K198 |  | 0.34 | 1.40E-03 |  |
|  |  |  | Altered Disordered interface |  | 0.36 | 8.90E-03 |  |
|  |  |  | Gain of Ubiquitylation at K197 |  | 0.19 | 0.01 |  |
|  |  |  | Loss of Methylation at K198 |  | 0.15 | 2.00E-02 |  |
|  |  |  | Altered Coiled coil |  | 0.14 | 3.00E-02 |  |
| **K205** | Lys(K)-Leu(L) | Non-polar | Loss of Acetylation at K205 | 0.529 | 0.58 | 3.60E-04 | ELME000106, ELME000146 |
|  |  |  | Altered Disordered interface |  | 0.39 | 7.30E-03 |  |
|  |  |  | Altered Coiled coil |  | 0.39 | 7.00E-03 |  |
|  |  |  | Loss of SUMOylation at K205 |  | 0.32 | 2.30E-03 |  |
|  |  |  | Loss of Methylation at K210 |  | 0.24 | 2.50E-03 |  |
|  |  |  | Altered DNA binding |  | 0.2 | 2.00E-02 |  |
|  |  |  | Loss of Ubiquitylation at K205 |  | 0.17 | 0.02 |  |
| **K210** | Lys(K)-Leu(L) | Non-polar | Loss of Acetylation at K210 | 0.833 | 0.79 | 7.80E-05 | ELME000008, PS00004 |
|  |  |  | Altered Coiled coil |  | 0.51 | 6.90E-03 |  |
|  |  |  | Altered Disordered interface |  | 0.46 | 3.90E-03 |  |
|  |  |  | Loss of Methylation at K210 |  | 0.41 | 1.80E-04 |  |
|  |  |  | Loss of B-factor |  | 0.31 | 5.30E-03 |  |
|  |  |  | Altered DNA binding |  | 0.3 | 2.70E-03 |  |
|  |  |  | Loss of Helix |  | 0.27 | 4.00E-02 |  |
|  |  |  | Loss of SUMOylation at K210 |  | 0.24 | 0.01 |  |
|  |  |  | Gain of Ubiquitylation at K205 |  | 0.16 | 0.03 |  |
|  | Lys(K)-Gln(Q) | Polar | Loss of Acetylation at K210 | 0.559 | 0.79 | 7.80E-05 | ELME000008, PS00004 |
|  |  |  | Loss of Methylation at K210 |  | 0.41 | 1.80E-04 |  |
|  |  |  | Altered Disordered interface |  | 0.38 | 7.60E-03 |  |

**S14**

|  |  |  | Altered Coiled coil |  | 0.3 | 1.00E-02 |  |
| --- | --- | --- | --- | --- | --- | --- | --- |
|  |  |  | Loss of B-factor |  | 0.29 | 1.00E-02 |  |
|  |  |  | Altered DNA binding |  | 0.27 | 6.30E-03 |  |
|  |  |  | Loss of SUMOylation at K210 |  | 0.24 | 0.01 |  |
|  |  |  | Gain of Ubiquitylation at K205 |  | 0.16 | 0.03 |  |
|  | Lys(K)-Glu(E) | Negatively | Loss of Acetylation at K210 | 0.686 | 0.79 | 7.80E-05 | ELME000008, PS00004 |
|  |  |  | Altered Disordered interface |  | 0.57 | 1.10E-03 |  |
|  |  |  | Loss of Methylation at K210 |  | 0.41 | 1.80E-04 |  |
|  |  |  | Altered Coiled coil |  | 0.36 | 8.30E-03 |  |
|  |  |  | Gain of Helix |  | 0.28 | 2.00E-02 |  |
|  |  |  | Loss of B-factor |  | 0.27 | 2.00E-02 |  |
|  |  |  | Altered DNA binding |  | 0.27 | 5.30E-03 |  |
|  |  |  | Gain of SUMOylation at K205 |  | 0.26 | 6.80E-03 |  |
|  |  |  | Gain of Ubiquitylation at K205 |  | 0.18 | 0.02 |  |
| **K211** | Lys(K)-Leu(L) | Non-polar | Loss of Acetylation at K211 | 0.683 | 0.54 | 4.50E-04 | ELME000008, PS00004 |
|  |  |  | Altered Coiled coil |  | 0.53 | 4.00E-03 |  |
|  |  |  | Loss of Methylation at K211 |  | 0.39 | 2.00E-04 |  |
|  |  |  | Altered Disordered interface |  | 0.38 | 6.70E-03 |  |
|  |  |  | Loss of B-factor |  | 0.33 | 2.90E-03 |  |
|  |  |  | Loss of Helix |  | 0.28 | 0.03 |  |
|  |  |  | Altered DNA binding |  | 0.28 | 4.90E-03 |  |
|  |  |  | Loss of SUMOylation at K211 |  | 0.27 | 4.50E-03 |  |
| **K215** | Lys(K)-Leu(L) | Non-polar | Loss of Acetylation at K215 | 0.817 | 0.64 | 2.10E-04 | ELME000008, PS00005 |
|  |  |  | Altered Coiled coil |  | 0.61 | 3.00E-03 |  |
|  |  |  | Altered DNA binding |  | 0.37 | 6.00E-04 |  |
|  |  |  | Altered Disordered interface |  | 0.33 | 0.01 |  |
|  |  |  | Loss of B-factor |  | 0.31 | 6.40E-03 |  |
|  |  |  | Loss of Helix |  | 0.28 | 0.03 |  |
|  |  |  | Loss of Methylation at K215 |  | 0.28 | 9.20E-04 |  |
|  |  |  | Gain of SUMOylation at K211 |  | 0.21 | 0.03 |  |
|  | Lys(K)-Gln(Q) | Polar | Loss of Acetylation at K215 | 0.576 | 0.64 | 2.10E-04 | ELME000008, PS00005 |
|  |  |  | Altered Coiled coil |  | 0.35 | 8.30E-03 |  |
|  |  |  | Altered DNA binding |  | 0.29 | 3.70E-03 |  |
|  |  |  | Loss of B-factor |  | 0.28 | 0.02 |  |
|  |  |  | Gain of Helix |  | 0.28 | 0.03 |  |
|  |  |  | Loss of Methylation at K215 |  | 0.28 | 9.20E-04 |  |
|  |  |  | Loss of SUMOylation at K215 |  | 0.2 | 0.03 |  |
| **K216** | Lys(K)-Leu(L) | Non-polar | Loss of Acetylation at K216 | 0.576 | 0.58 | 3.50E-04 | ELME000008, ELME000052, ELME000100, ELME000108, ELME000146 |
|  |  |  | Altered Coiled coil |  | 0.38 | 0.01 |  |
|  |  |  | Loss of Methylation at K216 |  | 0.37 | 2.40E-04 |  |
|  |  |  | Altered DNA binding |  | 0.35 | 9.00E-04 |  |
|  |  |  | Altered Disordered interface |  | 0.3 | 0.02 |  |
|  |  |  | Loss of B-factor |  | 0.3 | 1.00E-02 |  |
|  |  |  | Loss of Helix |  | 0.28 | 0.03 |  |

**S15**

|  |  |  | Gain of SUMOylation at K212 |  | 0.2 | 0.03 |  |
| --- | --- | --- | --- | --- | --- | --- | --- |
|  | Lys(K)-Gln(Q) | Polar | Loss of Acetylation at K216 | 0.639 | 0.58 | 3.50E-04 | ELME000008, ELME000100, ELME000108, ELME000146 |
|  |  |  | Loss of Methylation at K216 |  | 0.37 | 2.40E-04 |  |
|  |  |  | Altered Coiled coil |  | 0.32 | 9.40E-03 |  |
|  |  |  | Altered DNA binding |  | 0.29 | 3.60E-03 |  |
|  |  |  | Gain of Helix |  | 0.28 | 0.03 |  |
|  |  |  | Loss of B-factor |  | 0.27 | 2.00E-02 |  |
|  |  |  | Loss of SUMOylation at K211 |  | 0.2 | 0.03 |  |
|  | Lys(K)-Glu(E) | Negatively | Loss of Acetylation at K216 | 0.751 | 0.58 | 3.50E-04 | ELME000008, ELME000064, ELME000100, ELME000108, ELME000146, ELME000220, PS00006 |
|  |  |  | Loss of Methylation at K216 |  | 0.37 | 2.40E-04 |  |
|  |  |  | Altered Coiled coil |  | 0.32 | 9.50E-03 |  |
|  |  |  | Altered DNA binding |  | 0.31 | 2.10E-03 |  |
|  |  |  | Gain of Helix |  | 0.28 | 0.02 |  |
|  |  |  | Loss of B-factor |  | 0.26 | 4.00E-02 |  |
|  |  |  | Gain of SUMOylation at K211 |  | 0.23 | 0.02 |  |

* The pathogenic score in the table indicates the likelihood that the amino acid substitution is pathogenic. A score threshold of 0.50 would indicate that a specific substitution is pathogenic
